# Embryo‐Derived Cathepsin B Promotes Implantation and Decidualization by Activating Pyroptosis

**DOI:** 10.1002/advs.202402299

**Published:** 2024-09-24

**Authors:** Meng‐Yuan Li, Ying Wu, Hao‐Lan Tang, Ying Wang, Bo Li, Yu‐Ying He, Gui‐Jun Yan, Zeng‐Ming Yang

**Affiliations:** ^1^ Key Laboratory of Animal Genetics Breeding and Reproduction in the Plateau Mountain Region College of Animal Science Guizhou University Guiyang 550025 China; ^2^ College of Veterinary Medicine South China Agricultural University Guangzhou 510642 China; ^3^ Center for Reproductive Medicine and Obstetrics and Gynecology Nanjing Drum Tower Hospital Nanjing University Medical School Nanjing 210008 China

**Keywords:** implantation, inflammatory, pyroptosis

## Abstract

Embryo implantation and decidualization are crucial for a successful pregnancy. How the inflammatory response is regulated during these processes is undefined. Pyroptosis is an inflammatory form of cell death mediated by gasdermin D (GSDMD). Through in vivo, cultured epithelial cells and organoids, it is shown that pyroptosis occurs in epithelial cells at the implantation site. Compared with those on day 4 of pseudopregnancy and delayed implantation, pyroptosis‐related protein levels are significantly increased on day 4 of pregnancy and activated implantation, suggesting that blastocysts are involved in regulating pyroptosis. Blastocyst‐derived cathepsin B (CTSB) is stimulated by preimplantation estradiol‐17β and induces pyroptosis in epithelial cells. Pyroptosis‐induced IL‐18 secretion from epithelial cells activates a disintegrin and metalloprotease 12 (ADAM12) to process the epiregulin precursor into mature epiregulin. Epiregulin (EREG) enhances in vitro decidualization in mice. Pyroptosis‐related proteins are detected in the mid‐secretory human endometrium and are elevated in the recurrent implantation failure endometrium. Lipopolysaccharide treatment in pregnant mice causes implantation failure and increases pyroptosis‐related protein levels. Therefore, the data suggest that modest pyroptosis is beneficial for embryo implantation and decidualization. Excessive pyroptosis can be harmful and lead to pregnancy failure.

## Graphical abstract

The schematic view shows that embryo‐derived CTSB promotes implantation and decidualization by activating pyroptosis. Blastocyst‐derived CTSB activates NLRP3‐Caspase 1 to cleave GSDMD and cut pro‐IL‐1β/pro‐IL18 to form mature IL‐1β/IL‐18. The cleaved GSDMD‐N perforates the plasma membrane to release mature IL‐1β/IL‐18. IL‐18 promotes endometrial decidualization by stimulating ADAM12 and EREG in epithelial cells.

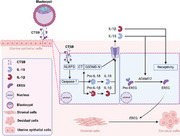

## 1 Introduction

Embryo implantation, a crucial step in mammalian reproduction, involves an intricate interaction between the receptive uterus and the competent blastocyst.^[^ ^1^ ^]^ In humans, ≈30% of successful natural pregnancies occur during each menstrual cycle, and 75% of unsuccessful pregnancies are due to failed implantation.^[^ ^2^ ^]^ Even if there are continuously great advances in assisted reproductive technology, ≈10% of women still suffer from recurrent implantation failure (RIF).^[^ ^3^ ^]^ RIF is defined as the transfer of high‐quality embryos that fail to implant after at least three in vitro fertilization (IVF) attempts.^[^ ^4^ ^]^ The reasons for unexplained RIF originate from the embryo and the endometrium, for example, poor quality embryos or disorders of endometrial function.^[^ ^4^ ^]^ A high level of C─C motif chemokine ligand 2, which disrupts the proinflammatory environment during embryo implantation, is detected in RIF patients.^[^ ^5^ ^]^ Uterine treatment with peripheral blood mononuclear cells can modulate the inflammatory response to improve the implantation rate in RIF patients.^[^ ^6^ ^]^ However, the underlying mechanism in the RIF endometrium is still unclear.

During embryo implantation, a temporary and modest proinflammatory response is beneficial for blastocyst implantation.^[^ ^7^ ^]^ Local injury to the endometrium can improve the implantation rate through inflammatory stimuli.^[^ ^8^ ^]^ Pregnant women taking nonsteroidal anti‐inflammatory drugs often fail in implantation, which is attributed to alterations in the inflammatory response during embryo implantation.^[^ ^9^ ^]^ Sterile inflammatory molecules, including high mobility group box 1 (HMGB1), ATP, and uric acid, are involved in decidualization.^[^ ^10^ ^]^ The favorable inflammatory environment during embryo implantation includes the recruitment and differentiation of immune cells, regeneration of the endometrium and angiogenesis, and endometrial cell differentiation.^[^ ^11^ ^]^ To date, it is still unclear how the inflammatory response is modulated during embryo implantation and decidualization.

Programmed cell death is necessary for organismal development and homeostasis.^[^ ^12^ ^]^ Cell death occurs in the inner cell mass of blastocysts, uterine epithelial cells, and stromal cells during embryo implantation and decidualization.^[^ ^13^ ^]^ There are six members of the gasdermin (GSDM) family in humans, including gasdermin A (GSDMA), gasdermin B (GSDMB), gasdermin C (GSDMC), gasdermin D (GSDMD), gasdermin E and pejvakin.^[^ ^14^ ^]^ Mice have no GSDMB but possess three GSDMAs (GSDMA1–3) and four GSDMCs (GSDMC1–4).^[^ ^15^ ^]^ Pyroptosis, an inherently inflammatory form, is defined as GSDMD‐mediated programmed necrosis.^[^ ^16^ ^]^ In canonical pyroptosis, stimuli from microbial infections or damaged cells are recognized by pattern recognition receptors to activate NOD‐like receptor (NLR) family pyrin domain‐containing 3 (NLRP3) oligomerization. Subsequently, oligomerized NLRP3 aggregates apoptosis‐associated speck‐like protein containing a CARD (ASC) and recruits procaspase 1 to form the NLRP3‐ASC‐caspase 1 protein complex, which is known as the NLRP3 inflammasome in mice and humans.^[^ ^17^ ^]^ Active caspase 1 cleaves GSDMD to release the N‐terminal domain (GSDMD‐N) and cut pro‐IL‐1β/pro‐IL18 to form mature IL‐1β/IL‐18.^[^ ^18^ ^]^ Cleaved GSDMD‐N perforates the plasma membrane to form 10–15 nm pores, resulting in inflammatory cell death pyroptosis. Pyroptosis causes the release of mature IL‐1β/IL‐18.^[^ ^19^ ^]^ The non‐canonical pyroptosis is mediated by active mouse caspase 11 and its human orthologues caspase 4 and caspase 5.^[^ ^18^ ^]^ With the release of inflammatory cytokines and cellular contents, pyroptosis causes the activation and further expansion of the inflammatory response.^[^ ^19^ ^]^ Accumulating evidence indicates that pyroptosis leads to the release of HMGB1, ATP, IL‐18, and IL‐1β,^[^ ^20^ ^]^ which are involved in embryo implantation and decidualization.^[^ ^10^ ^]^ Therefore, we suggest that inflammation induced by pyroptosis plays a role in embryo implantation and decidualization.

In this study, we analyzed the regulation of pyroptosis during embryo implantation and decidualization in mice. Pyroptosis‐related protein levels are significantly increased in the luminal epithelium at the time of embryo implantation. Blastocyst‐derived cathepsin B (CTSB) activates pyroptosis and IL‐18 secretion, which enhances stromal cell decidualization through activating epithelial a disintegrin and metalloprotease 12 (ADAM12) and epiregulin (EREG).

## 2 Results

### 2.1 Pyroptosis in Day 4 Pregnant Mouse Uterus

**Figure 1:**
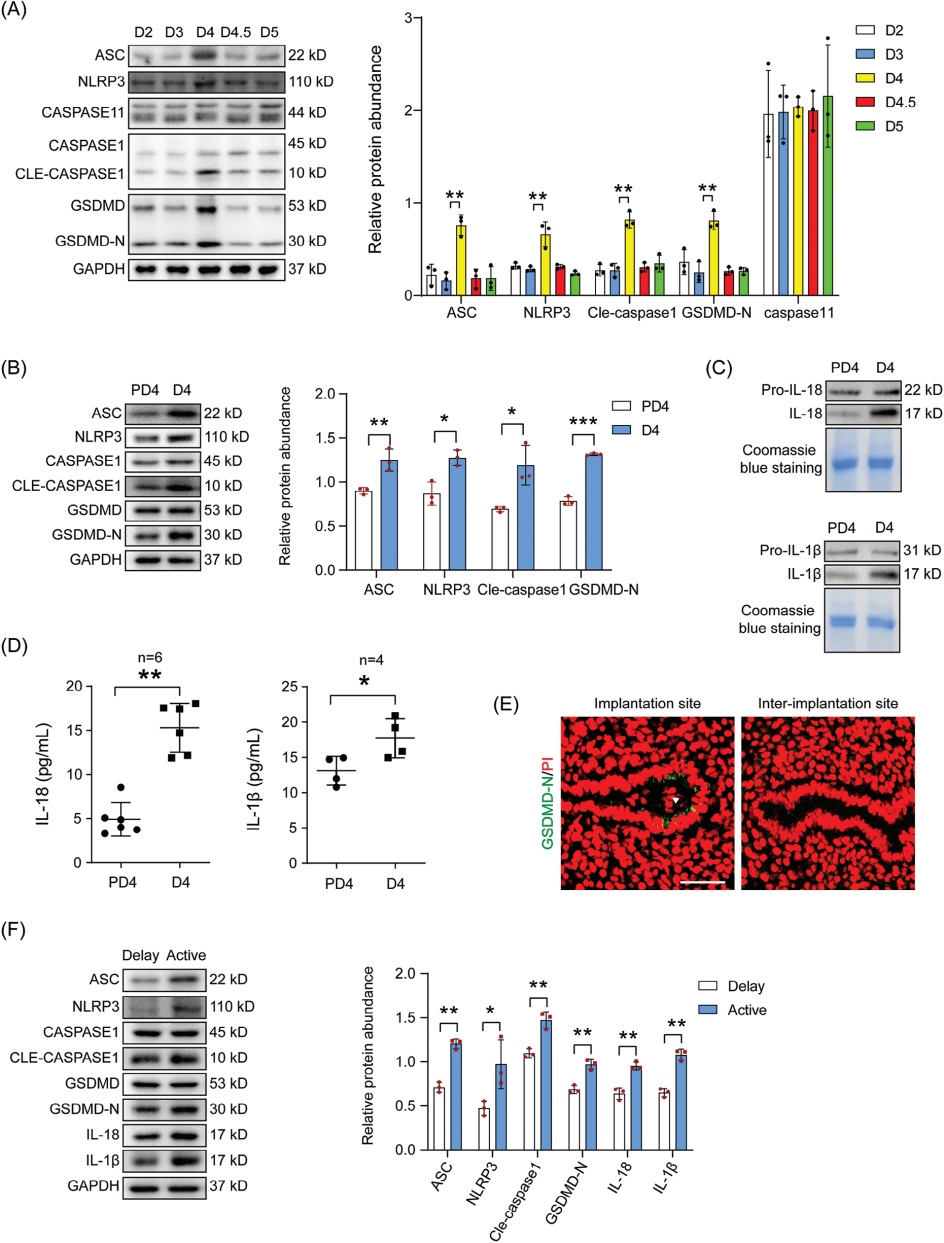
Pyroptosis in day 4 pregnant mouse uterus. A) Western blot analysis and quantification of ASC, NLRP3, CASPASE 11, CASPASE 1, cleaved CASPASE 1, GSDMD‐N, and GSDMD proteins levels on days 2, 3, 4, 4.5 and 5 of pregnancy (*n* = 3 per group). B) Western blot analysis and quantification of ASC, NLRP3, CASPASE 1, cleaved CASPASE 1, GSDMD‐N, and GSDMD in mouse uteri on day 4 of pregnancy and day 4 of pseudopregnancy (*n* = 3 per group). C) Western blot analysis of IL‐18 and IL‐1β levels in uterine luminal fluid on day 4 of pregnancy and day 4 of pseudopregnancy. Coomassie brilliant blue staining of uterine luminal fluid proteins was used for loading control. D) ELISA analysis of IL‐18 and IL‐1β concentrations in uterine luminal fluid on day 4 of pregnancy and day 4 of pseudopregnancy. n, the number of samples. E) GSDMD‐N immunofluorescence (green) and PI fluorescence (red) at the implantation site and inter‐implantation site on day 4.5 of pregnancy. Arrowhead, blastocyst. F) Western blot and quantification analysis of ASC, NLRP3, CASPASE 1, cleaved CASPASE 1, GSDMD‐N, GSDMD, IL‐18, and IL‐1β protein levels under delayed and activated implantation (*n* = 3 per group). Scale bar = 250 µm. Data were presented as mean ± SD. ^*^: *p* < 0.05; ^**^: *p* < 0.01; ^***^: *p* < 0.001, by two‐tailed Student's t‐test.

There are two main pathways of pyroptosis, the caspase 1‐dependent classical pathway and the caspase 11‐dependent nonclassical pathway.^[^ ^21^ ^]^ Western blot and quantitative analysis revealed that the uterine levels of cleaved CASPASE 1, ASC, NLRP3, and GSDMD‐N on day 4 of pregnancy were greater than those on days 2, 3, 4.5, and 5 of pregnancy (**Figure** **1**A). However, the caspase 11 level did not change during early pregnancy (Figure 1A), suggesting that the caspase 1‐dependent classical pathway is the dominant pathway. To further analyze the effects of embryos on pyroptosis, pyroptosis‐related protein levels were compared between day 4 of pregnancy and day 4 of pseudopregnancy. Compared with those on day 4 of pseudopregnancy, the levels of the proteins NLRP3, ASC, cleaved caspase 1, and GSDMD‐N increased on day 4 of pregnancy (Figure 1B). Because pyroptosis is accompanied by apoptosis and necroptosis,^[^ ^22^ ^]^ we examined apoptosis by cleaved CASPASE 3 and necroptosis by phosphorylated mixed lineage kinase domain‐like protein (MLKL) and protein kinase receptor‐interacting protein 3 (RIP3) in mouse uterus on day 4 of pregnancy.^[^ ^23^ ^]^ Although cleaved caspase 3 and phosphorylated MLKL were detected in positive tissues (Figure S1A, Supporting Information), the signals for cleaved CASPASE 3, phosphorylated MLKL, and phosphorylated RIP3 were not seen in day 4 pregnant uterus (Figure S1A–D, Supporting Information).

Both ELISA and Western blot showed that the protein levels of uterine IL‐18 and IL‐1β were also increased on day 4 of pregnancy compared with those on day 4 of pseudopregnancy (Figure 1C,D). GSDMD‐N immunofluorescence was seen in the luminal epithelium surrounding the implanted blastocyst on day 4.5 of pregnancy (Figure 1E).

To further analyze whether pyroptosis is dependent on active blastocysts, pyroptosis‐related protein levels were compared between delayed and activated implantation. Western blot analysis and quantification revealed that the protein levels of NLRP3, ASC, cleaved CASPASE 1, GSDMD‐N, IL‐18, and IL‐1β were significantly increased after delayed implantation was activated by estradiol‐17β (Figure 1F). These results further revealed that the occurrence of pyroptosis is dependent on the presence of blastocysts.

### 2.2 Blastocyst‐Derived CTSB Triggers Pyroptosis

**Figure 2:**
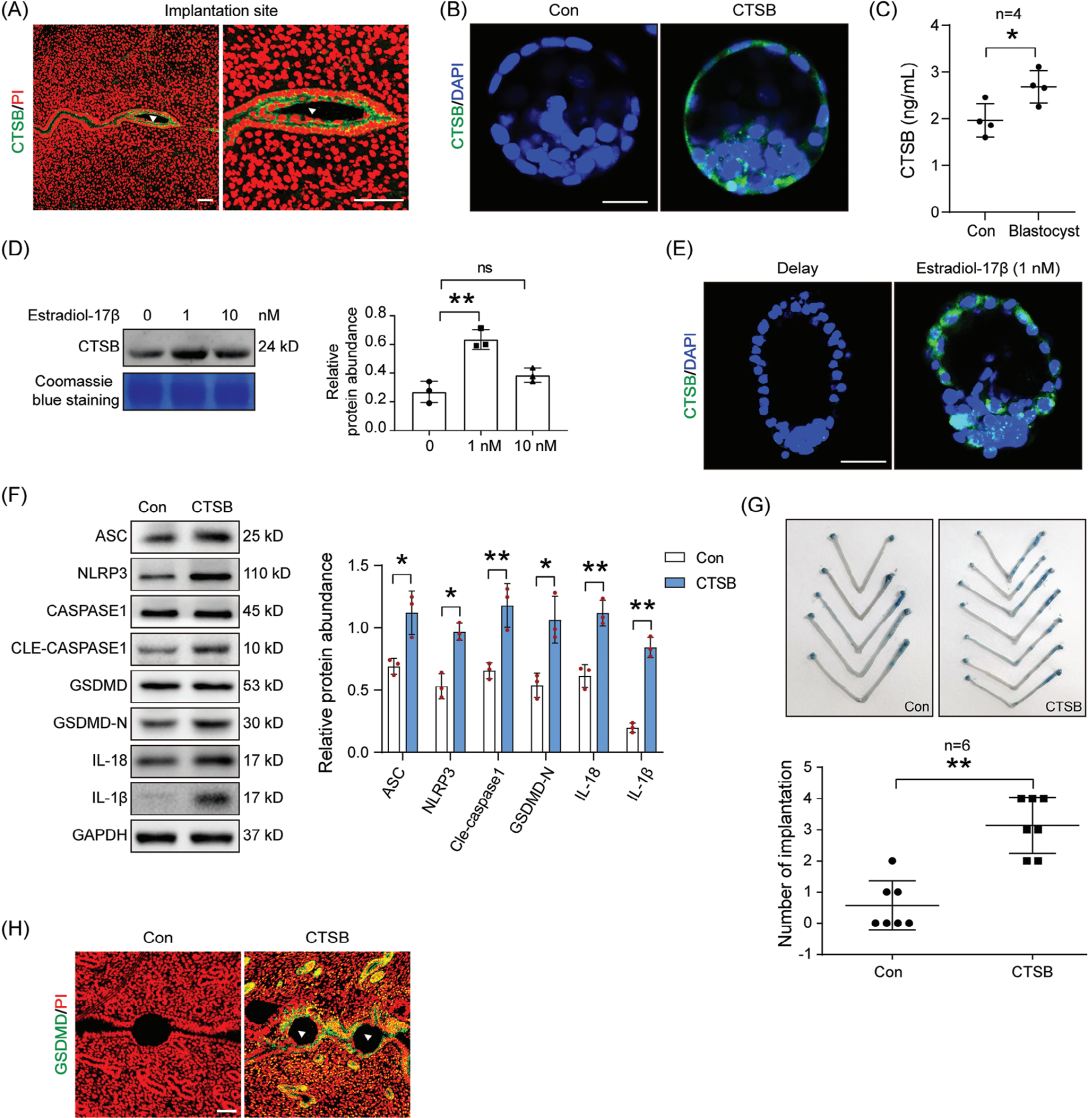
Blastocyst‐derived CTSB triggers pyroptosis. A) CTSB immunofluorescence (green) and PI fluorescence (red) at the implantation site on day 5 of pregnancy. Arrowhead, blastocyst. B) CTSB immunofluorescence of CTSB (green) and DAPI fluorescence (blue) in mouse blastocyst collected on day 4 of pregnancy. C) ELISA analysis of CTSB protein in the cultured medium after blastocytes were cultured in KSOM medium for 6 h (*n* = 4 per group). D) Western blot analysis and quantification of CTSB protein levels in cultured medium after dormant blastocysts were treated with different concentrations of estradiol‐17β for 12 h (*n* = 3 per group). Coomassie bright blue staining was used as a loading control. E) CTSB immunofluorescence (green) and DAPI fluorescence (blue) in dormant and dormant blastocysts treated with 1 nM estradiol‐17β for 12 h. F) Western blot analysis and quantification of uterine ASC, NLRP3, CASPASE 1, cleaved CASPASE 1, GSDMD‐N, GSDMD, IL‐18, and IL‐1β protein levels after CTSB was injected into uterine lumen of day 4 pseudopregnant mice (*n* = 3 per group). G) The blue bands of attachment response after CTSB‐soaked beads were transferred into the uterine lumen of day 4 pseudopregnant mice for 24 h (*n* = 6 per group). H) GSDMD immunofluorescence (green) and PI fluorescence (red) after CTSB‐soaked beads were transferred into the uterine lumen of day 4 pseudopregnant mice. Arrowhead, CTSB‐soaked beads. Scale bar = 125 µm. Data were presented as mean ± SD. ^*^: *p* < 0.05; ^**^: *p* < 0.01; ns: not significant, by two‐tailed Student's t‐test.

Because our data showed that pyroptosis is dependent on active blastocysts, we further explored how blastocysts induce pyroptosis in the luminal epithelium. Mouse blastocysts can synthesize and secrete S100 calcium binding protein A9 (S100A9) and tumor necrosis factor (TNF).^[^ ^24^ ^]^ When mouse epithelial cells were treated with S100A9 or TNF, pyroptosis‐related protein levels had no clear changes (Figure S2A,B, Supporting Information). When mouse epithelial cells were treated with S100A9 or TNF, pyroptosis‐related protein levels had no clear changes (Figure S2A,B, Supporting Information). CTSB is increased in mouse activated blastocysts and is also involved in pyroptosis.^[^ ^24^, ^25^ ^]^ CTSB immunofluorescence was observed in the luminal epithelium and implanting blastocyst at the implantation site on the day of pregnancy (**Figure** **2**A). In mouse blastocysts, CTSB immunofluorescence was mainly localized in trophoblastic cells (Figure 2B). The secretion of CTSB protein in the culture medium was increased after mouse blastocysts were cultured for 6 h (Figure 2C; Figure S2C, Supporting Information). After dormant blastocysts were treated with different concentrations of estradiol‐17β for 12 h, the CTSB level in the culture medium was also increased (Figure 2D). Compared with that in delayed blastocysts, CTSB immunofluorescence was highly detected in activated blastocyst 12 h after delayed implantation was activated by estradiol‐17β (Figure S2D, Supporting Information). Additionally, CTSB immunofluorescence was also increased after the delayed blastocyst was treated with 1 nM estradiol‐17β (Figure 2E). When CTSB was injected into the uterine lumen in the morning on day 4 of pseudopregnancy, pyroptosis‐related protein levels were increased (Figure 2F). After the CTSB‐soaked beads were transferred into the uterine lumen on day 4 of pseudopregnancy, blue bands of the attachment response were also visualized (Figure 2G). HAND2 and cyclooxygenase 2 (COX2) are markers of decidualization in mice.^[^ ^1^ ^]^ HAND2 and COX2 immunofluorescence was seen in endometrial stroma cells surrounding CTSB‐soaked beads (Figure S2E, Supporting Information). Furthermore, after the CTSB‐soaked beads were transferred into the uterine horns of day 4 pseudopregnant mice, GSDMD immunofluorescence was detected in the luminal epithelium surrounding CTSB‐soaked beads (Figure 2H). These results further indicated that blastocyst‐derived CTSB was able to initiate pyroptosis in the luminal epithelium.

### 2.3 CTSB Induces Rapid and Reversible Pyroptosis in Mouse Endometrial Epithelial Cells

**Figure 3:**
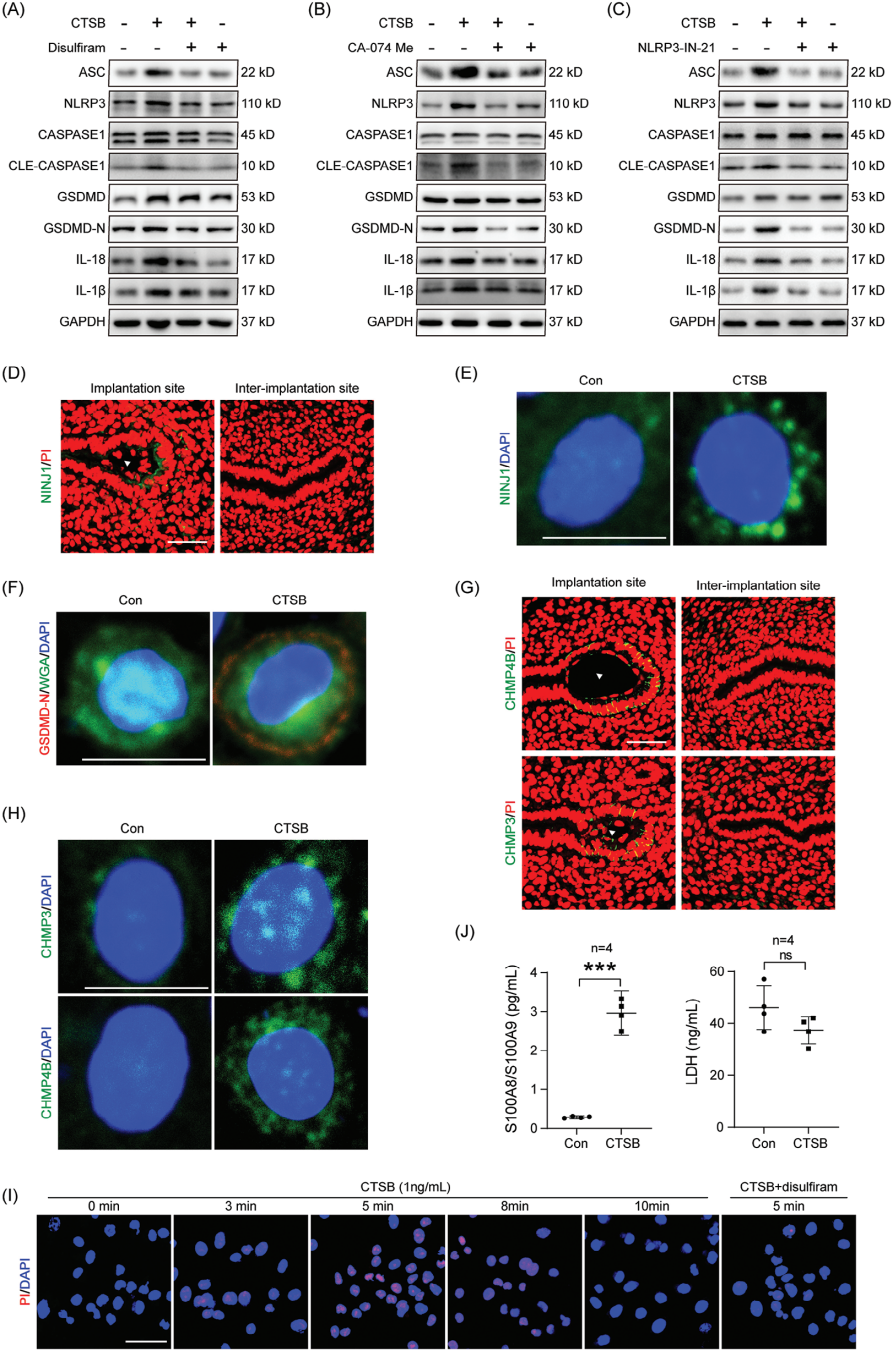
CTSB induces rapid and reversible pyroptosis in mouse endometrial epithelial cells. A) Western blot analysis of pyroptosis‐associated protein levels after endometrial epithelial cells were treated with CTSB, CTSB and disulfiram, or disulfiram for 3 h. B) Western blot analysis of pyroptosis‐associated protein levels after endometrial epithelial cells were treated with CTSB, CTSB and CA‐074 Me, or CA‐074 Me for 3 h. C) Western blot analysis of pyroptosis‐associated protein levels after endometrial epithelial cells were treated with CTSB, CTSB and NLRP3‐IN‐21, or NLRP3‐IN‐21 for 3 h. D) NINJ1 immunofluorescence (Green) and PI fluorescence (red) at the implantation site and inter‐implantation site on day 4.5 of pregnancy. Arrowhead, blastocyst. E) NINJ1 immunofluorescence (green) and DAPI fluorescence (blue) after endometrial epithelial cells were treated with CTSB for 5 min. F) GSDMD‐N immunofluorescence (red), WGA fluorescence (green), and DAPI fluorescence (blue) after endometrial epithelial cells were treated with CTSB for 5 min. G) CHMP4B and CHMP3 immunofluorescence (green) and PI fluorescence (red) at the implantation site and inter‐implantation site on day 4.5 of pregnancy. Arrowhead, blastocyst. H) CHMP3 and CHMP4B immunofluorescence (green) and DAPI fluorescence (blue) after endometrial epithelial cells were treated with CTSB for 10 min. I) PI fluorescence (red) and DAPI fluorescence (blue) after endometrial epithelial cells were treated with CTSB for different time points. J) ELISA analysis of S100A8/S100A9 and LDH secretion after endometrial epithelial cells were treated with CTSB for 5 min (*n* = 4 per group). Scale bar = 125 µm. Data were presented as mean ± SD.^***^: *p* < 0.001, ns: not significant, by two‐tailed Student's t‐test.

In cultured epithelial cells, the CTSB‐induced increase in pyroptosis‐related protein levels was abrogated by disulfiram (an inhibitor of pyroptosis), CA‐074 Me (an inhibitor of CTSB) and NLRP3‐IN‐21 (an inhibitor of NLRP3), respectively (**Figure** **3**A–C; Figure S3A–C, Supporting Information). Furthermore, mouse endometrial epithelial organoids were used to confirm the results from cultured epithelial cells. The CTSB‐stimulated increase of pyroptosis‐related protein levels in epithelial organoids was also suppressed by disulfiram, CA‐074 Me, and NLRP3‐IN‐21, respectively (Figure S3D–F, Supporting Information). When cultured epithelial cells transfected with pBOB‐mGSDMD‐NT‐Flag plasmid were treated with CTSB, GSDMD immunofluorescence increased, which was abrogated by disulfiram (Figure S3G, Supporting Information). Similarly, CTSB‐induced GSDMD immunofluorescence in epithelial organoids was suppressed by disulfiram (Figure S3H, Supporting Information).

Ninjurin1 (NINJ1) is activated downstream of GSDMD pore formation and oligomerizes into filamentous assemblies for disrupting membranes and forming pores.^[^ ^26^ ^]^ NINJ1 immunofluorescence was seen in the luminal epithelium at the implantation site compared to the inter‐implantation site (Figure 3D). The stimulation of CTSB caused the aggregation of NINJ in endometrial epithelial cells (Figure 3E). When epithelial cells were treated with CTSB, GSDMD‐N immunofluorescence was also co‐localized with the plasma membrane marker wheat germ agglutinin (WGA) at the surface of epithelial cells (Figure 3F). Charged multivesicular body protein 4B (CHMP4B) and charged multivesicular body protein 3 (CHMP3) are members of the endosomal sorting complex required for transport (ESCRT) machinery, which targets wound membranes to form a punctate pattern by removing GSDMD pores from the plasma membrane, and counterbalance cell death.^[^ ^27^ ^]^ Compared to the inter‐implantation site, CHMP4B and CHMP3 immunofluorescence was observed in the luminal epithelium surrounding the implanting blastocyst on day 4.5 of pregnancy (Figure 3G). Treatment with CTSB also induced the punctated assembly of CHMP4B and CHMP3 proteins in endometrial epithelial cells (Figure 3H).

Ethidium Homodimer III (EthD‐III), Propidium iodide (PI), and DRAQ7 are fluorescent nucleic acid dyes that can enter the cell to stain nuclear DNA when the integrity of the membrane is damaged, and are usually used to detect pyroptosis.^[^ ^23^, ^28^ ^]^ After epithelial cells were treated with CTSB, the PI fluorescence signal appeared at 3 min, reached the strongest level at 5 min, and began to decline from 8 min. PI signal detected 5 min after CTSB treatment was blocked by pretreatment with disulfiram (Figure 3I). The fluorescence signal pattern for EthD‐III and DRAQ7 was similar to PI after epithelial cells were treated with CTSB (Figure S3I, Supporting Information). S100A8/S100A9 can be released from GSDMD pores,^[^ ^28a^ ^]^ but lactate dehydrogenase (LDH) is too large to exit through GSDMD pores and acts as a marker for lytic cell death.^[^ ^28a^ ^]^ After epithelial cells were treated with CTSB for 5 min, S100A8/S100A9 secretion in the culture medium was significantly increased, but LDH secretion did not show an obvious change (Figure 3J). Based on these data, our results suggested that CTSB induced pyroptosis in mouse endometrial epithelial cells in a transient and reversible pattern.

### 2.4 Function of Pyroptosis during Embryo Implantation

**Figure 4:**
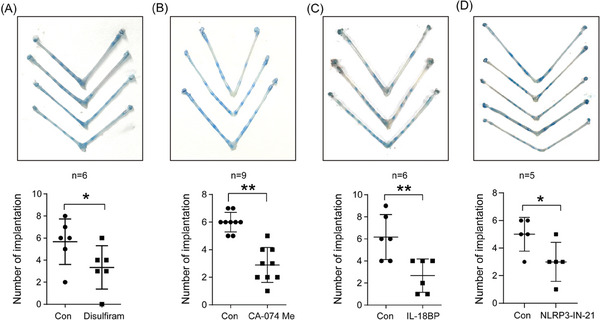
Function of pyroptosis during embryo implantation. A) The number of implantation sites on day 5 of pregnancy after disulfiram, an inhibitor of pyroptosis, was injected into the uterine lumen of day 4 pregnant mice (*n* = 6 per group). B) The number of implantation sites on day 5 of pregnancy after CA‐074 Me, an inhibitor of CTSB, was injected into the uterine lumen of day 4 pregnant mice (*n* = 9 per group). C) The number of implantation sites on day 5 of pregnancy after IL‐18BP was injected into the uterine lumen of day 4 pregnant mice (*n* = 6 per group). D) The number of implantation sites on day 5 of pregnancy after NLRP3‐IN‐21 was injected into the uterine lumen of day 4 pregnant mice (*n* = 5 per group). Data were presented as mean ± SD. ^*^: *p* < 0.05; ^**^: *p* < 0.01, by two‐tailed Student's t‐test.

Our results demonstrated that pyroptosis occurs in the luminal epithelium on day 4 of pregnancy. We further explored the function of pyroptosis during embryo implantation. When disulfiram, an FDA‐approved inhibitor for pyroptosis,^[^ ^29^ ^]^ was injected into the uterine horns of day 4 pregnant mice, the number of implantation sites was significantly reduced (**Figure** **4**A). The intrauterine injection of CA‐074 Me, an inhibitor of CTSB, also significantly decreased the number of implantation sites (Figure 4B). IL‐18 binding protein (IL‐18BP) acts as a natural inhibitor for IL‐18 by binding mature IL‐18.^[^ ^30^ ^]^ The number of implantation sites was significantly reduced by the intrauterine injection of mouse IL‐18BP (Figure 4C). Intrauterine injection of NLRP3‐IN‐21 (an inhibitor of NLRP3) also significantly decreased the number of implantation sites (Figure 4D).

### 2.5 Effects of IL‐18/IL‐1β on Endometrial Receptivity and Embryo Adhesion

**Figure 5:**
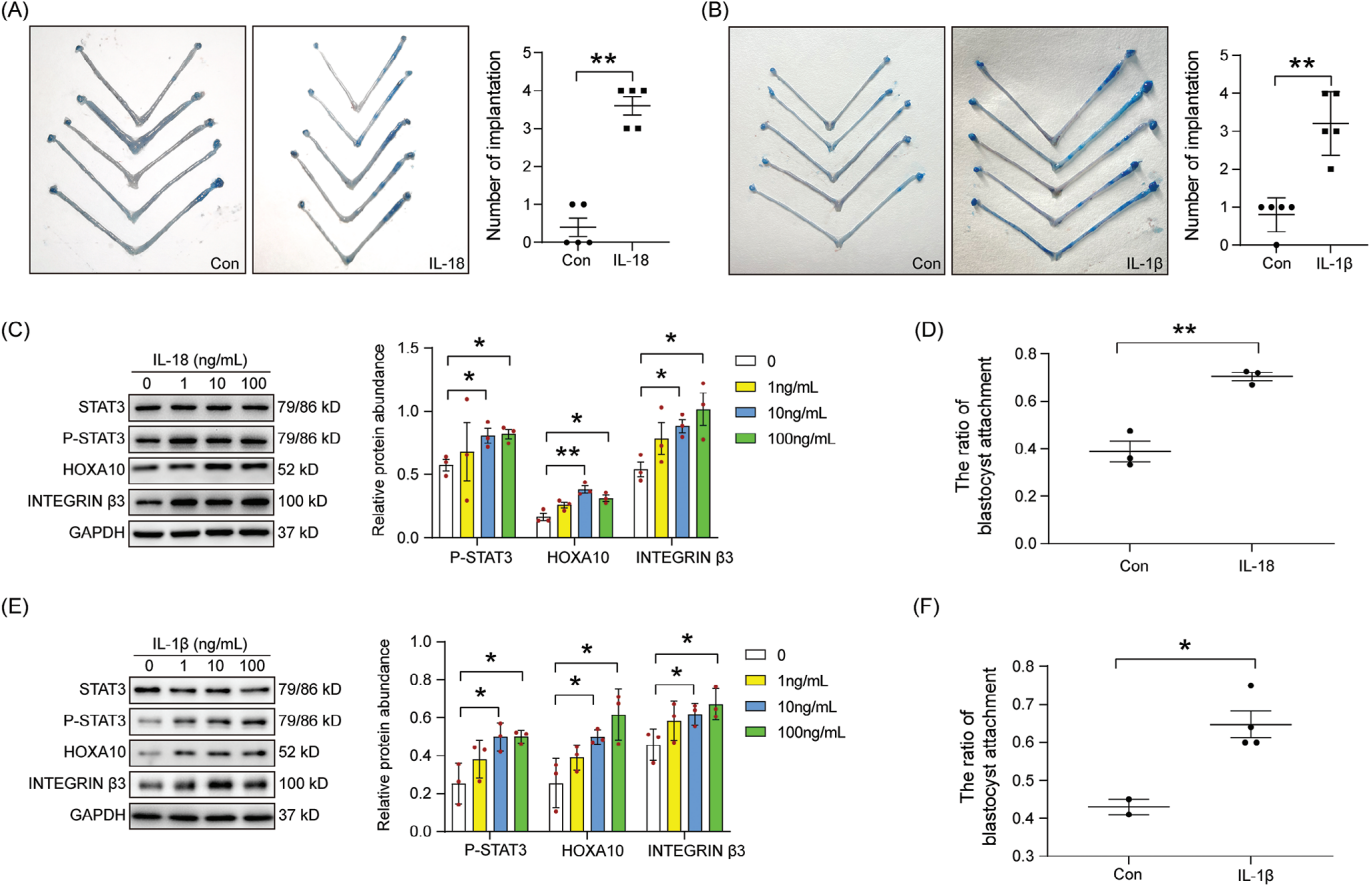
Effects of IL‐18 and IL‐1β on endometrial receptivity and embryo adhesion. A) The number of blue bands for attachment response after IL‐18‐soaked beads were transferred into the uterine lumen of day 4 pseudopregnant mice for 24 h (*n* = 5 per group). B) The number of blue bands after IL‐1β‐soaked beads were transferred into the uterine lumen of day 4 pseudopregnant mice for 24 h (*n* = 5 per group). C) Western blot and quantification analysis of p‐STAT3, HOXA10, and integrin β3 protein levels after endometrial epithelial cells were treated with different concentrations of IL‐18 for 3 h (*n* = 3 per group). D) The ratio of blastocyst attachment after blastocysts were cultured onto epithelial cells and treated with 10 ng mL^−1^ IL‐18 for 48 h (*n* = 3 per group). E) Western blot analysis and quantification for p‐STAT3, HOXA10, and integrin β3 protein levels after endometrial epithelial cells were treated with different concentrations of IL‐1β for 3 h (*n* = 3 per group). F) The ratio of blastocyst attachment after blastocysts were cultured onto epithelial cells and treated with 10 ng mL^−1^ IL‐1β for 48 h (*n* = 3 per group). Data were presented as mean ± SD. ^*^: *p* < 0.05; ^**^: *p* < 0.01, by two‐tailed Student's t‐test.

To further examine how pyroptosis contributes to embryo implantation, we analyzed the effects of pyroptosis downstream molecules on embryo implantation. After IL‐18‐soaked beads or IL‐1β‐soaked beads were injected into the uterine horn of day 4 pseudopregnant mice, the number of blue bands associated with the attachment reaction significantly increased (**Figure** **5**A,B). These observations further indicate that pyroptosis occurs during the window of implantation and participates in embryo implantation.

Homeobox A10 (HOXA10), integrin β, and phosphorylated signal transducer and activator of transcription 3 (p‐STAT3) are recognized markers of uterine receptivity.^[^ ^31^ ^]^ When cultured epithelial cells were treated with IL‐18, there was a dose‐dependent increase in the HOXA10, integrin β3, and p‐STAT3 protein levels (Figure 5C). After mouse blastocysts were co‐cultured on cultured epithelial cells, the attachment rate of blastocysts to epithelial cells was significantly increased by IL‐18 (Figure 5D). Similarly, the protein levels of p‐STAT3, HOXA10, and integrin β3 in epithelial cells were increased by IL‐1β (Figure 5E). IL‐1β also significantly enhanced the attachment rate of blastocysts on cultured epithelial cells (Figure 5F).

### 2.6 IL‐18 Promotes Decidualization via ADAM12/EREG

**Figure 6:**
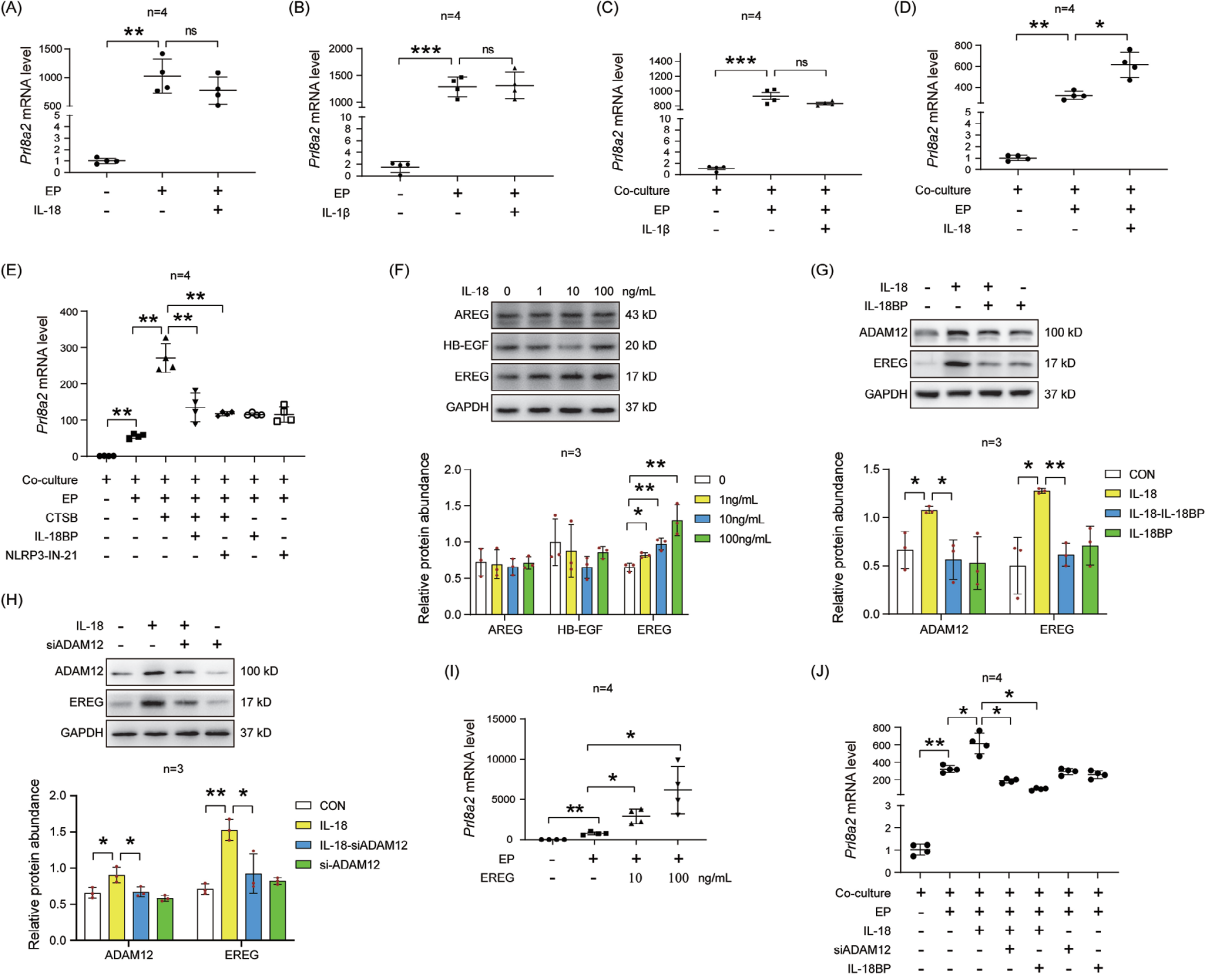
IL‐18 promotes decidualization through activating epithelial ADAM12 and EREG. A) qPCR analysis on effects of IL‐18 on *Prl8a2* mRNA level under in vitro decidualization for 48 h (*n* = 4 per group). B) qPCR analysis on effects of IL‐1β on *Prl8a2* mRNA level under in vitro decidualization for 48 h (*n* = 4 per group). C) qPCR analysis on *Prl8a2* mRNA level in stromal cells after the co‐culture of epithelial and stromal cells were treated with IL‐1β for 48 h (*n* = 4 per group). D) qPCR analysis on *Prl8a2* mRNA level in stromal cells after the co‐culture of epithelial and stromal cells were treated with IL‐18 for 48 h (*n* = 4 per group). E) qPCR analysis on *Prl8a2* mRNA level after the co‐culture of epithelial and stromal cells were treated with CTSB, CTSB and IL‐18BP, CTSB and NLRP3‐IN‐21, IL‐18BP, or NLRP3‐IN‐21 for 48 h (*n* = 4 per group). F) Western blot analysis and quantification of AREG, HB‐EGF, and EREG protein levels after endometrial epithelial cells were treated with IL‐18 for 3 h (*n* = 3 per group). G) Western blot analysis and quantification of ADAM12 and EREG protein levels after endometrial epithelial cells were treated with IL‐18, IL‐18 and IL‐18BP, or IL‐18BP for 3 h (*n* = 3 per group). H) Western blot analysis and quantification of ADAM12 and EREG protein levels after endometrial epithelial cells were treated with IL‐18, IL‐18 and ADAM12 siRNA, or ADAM12 siRNA for 3 h (*n* = 3 per group). I) qPCR analysis on *Prl8a2* mRNA level after stromal cells were treated with EREG under in vitro decidualization for 48 h (*n* = 4 per group). J) qPCR analysis on *Prl8a2* mRNA level after the co‐culture of epithelial and stromal cells under in vitro decidualization was treated with IL‐18, ADAM12 siRNA, IL‐18BP, IL‐18 and ADAM12 siRNA, or IL‐18 and IL‐18BP for 48 h (*n* = 4 per group). EP, treatment with estradiol‐17β and progesterone for in vitro decidualization. Data were presented as mean±SD. ^*^: *p* < 0.05; ^**^: *p* < 0.01; ^***^: *p* < 0.001; ns: not significant, by two‐tailed Student's t‐test.

To further explore whether epithelial IL‐18/IL‐1β promotes mouse decidualization, we tested the effects of IL‐18/IL‐1β on in vitro decidualization in mice. Prolactin family 8, subfamily A, member 2 (*Prl8a2*), Prolactin family 3, subfamily C, member 1 (*Prl3c1*), and *E2f8* are markers of mouse in vitro decidualization.^[^ ^32^ ^]^ When endometrial stromal cells were treated with IL‐18 or IL‐1β, the mRNA levels of *Prl8a2*, *Prl3c1*, and *E2f8* did not change (**Figure** **6**A,B; Figure S4A–D, Supporting Information). *Prl8a2*, *Prl3c1*, and *E2f8* mRNA levels in stromal cells also did not significantly increase when the co‐culture of epithelial and stromal cells were treated with IL‐1β (Figure 6C; Figure S4E,F, Supporting Information). However, *Prl8a2*, *Prl3c1*, and *E2f8* mRNA levels were significantly increased after the co‐culture of epithelial and stromal cells was treated with IL‐18 (Figure 6D; Figure S4G,H, Supporting Information). Furthermore, the CTSB‐induced increase in *Prl8a2*, *Prl3c1*, and *E2f8* mRNA levels in stromal cells was suppressed when the co‐culture of epithelial and stromal cells was treated by IL‐18BP or NLRP3‐IN‐21 (Figure 6E; Figure S4I,J, Supporting Information). These data suggest that IL‐18 might stimulate endometrial decidualization through epithelial‐stromal cross‐talk.

Epidermal growth factor (EGF) family members, including heparin‐binding epidermal growth factor (HB‐EGF), amphiregulin (AREG), and EREG, play important roles in the interaction between the embryo and uterus.^[^ ^33^ ^]^ IL‐1β, IL‐6, and IL‐17A promote the expression of EREG.^[^ ^34^ ^]^ In our study, IL‐18, but not IL‐1β, promoted decidualization by co‐culture of epithelial and stromal cells in mice. Therefore, we speculated that IL‐18 participates in decidualization mediated by EGF family members. When epithelial cells were treated with mouse IL‐18 protein, the protein levels of EREG significantly changed, whereas AREG and HB‐EGF protein levels did not significantly change (Figure 6F). EREG immunofluorescence was detected in the uterine epithelium and decidual cells at the implantation site (Figure S4K, Supporting Information). ADAM12, a member of a disintegrin and metalloprotease family (ADAM), mediates proteolytic maturation of the precursors of EGF family members.^[^ ^35^ ^]^ ADAM12 immunofluorescence was observed in the luminal epithelium and decidual cells at the implantation site (Figure S4K, Supporting Information). When cultured epithelial cells were treated with IL‐18, the protein levels of EREG and ADAM12 increased, which was abrogated by IL‐18BP (Figure 6G). ADAM12 knockdown also suppressed the IL‐18‐induced increase in EREG and ADAM12 protein levels in epithelial cells (Figure 6H). Under in vitro decidualization, EREG significantly increased *Prl8a2*, *Prl3c1*, and *E2f8* mRNA levels in stromal cells (Figure 6I; Figure S4L,M, Supporting Information). When epithelial cells were cocultured with stromal cells, the IL‐18‐induced increase in *Prl8a2*, *Prl3c1*, and *E2f8* mRNA levels were repressed by IL‐18BP or ADAM12 siRNA (Figure 6J; Figure S4N,O, Supporting Information). These findings suggest that IL‐18 might promote endometrial decidualization by stimulating ADAM12 and EREG in epithelial cells.

### 2.7 LPS Leads to Abortion Through Excess Activation of Pyroptosis

**Figure 7:**
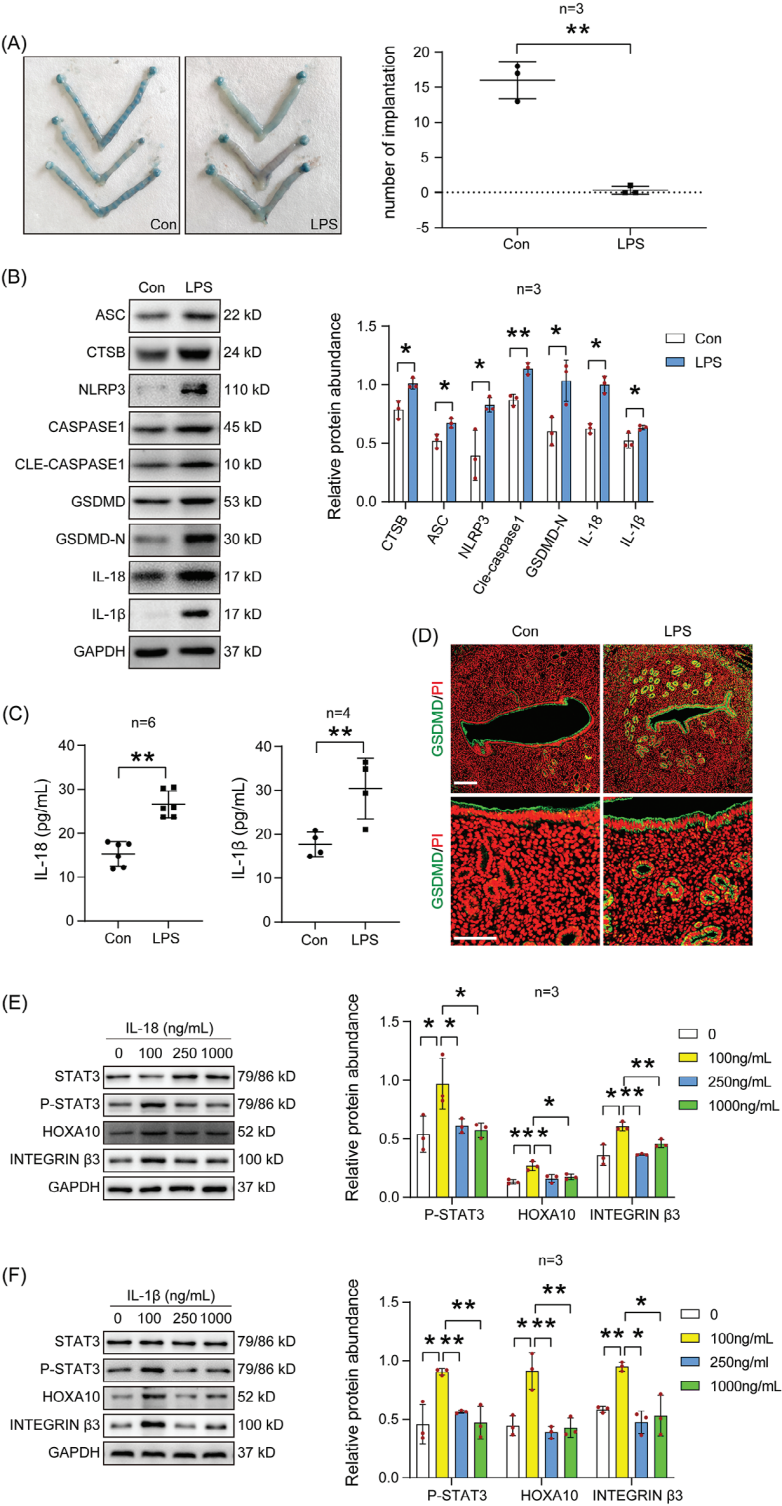
LPS leads to abortion through excess activation of pyroptosis. A) The number of implantation sites on day 5 of pregnancy after day 3 pregnant mice were treated with LPS (*n* = 3 per group). B) Western blot analysis and quantification of pyroptosis‐associated protein levels in LPS‐treated mouse uterus (*n* = 3 per group). C) ELISA analysis of IL‐18 and IL‐1β concentrations in uterine luminal fluid of LPS‐treated mouse uterus (*n* = 4–6 per group). D) GSDMD immunofluorescence (green) and PI fluorescence (red) in LPS‐treated mouse uterus. E) Western blot analysis and quantification of p‐STAT3, HOXA10, and integrin β3 protein levels after endometrial epithelial cells were treated with different concentrations of IL‐18 protein for 3 h (*n* = 3 per group). F) Western blot analysis and quantification of p‐STAT3, HOXA10, and integrin β3 protein levels after endometrial epithelial cells were treated with different concentrations of IL‐1β protein for 3 h (*n* = 3 per group). Data were presented as mean ± SD. ^*^: *p* < 0.05; ^**^: *p* < 0.01, by two‐tailed Student's t‐test.

LPS, a gram‐negative bacterial lipopolysaccharide that can cause inflammation, is used to induce implantation loss in mice by causing an excessive inflammatory response.^[^ ^36^ ^]^ LPS can induce pyroptosis in acute lung injury models and human gingival fibroblasts.^[^ ^37^ ^]^ When female mice were intraperitoneally injected with LPS to induce implantation loss on day 3 of pregnancy as previously described,^[^ ^36^ ^]^ the number of implantation sites on day 5 of pregnancy was significantly reduced (**Figure** **7**A). Wingless‐related murine mammary tumor virus integration site 4 (WNT4), bone morphogenetic protein 2 (BMP2), and COX2 are markers of decidualization.^[^ ^1^ ^]^ Compared with the strong immunofluorescence in the control, WNT4, BMP2, and COX2 immunofluorescence was barely detected in LPS‐treated mouse uteri (Figure S5A, Supporting Information). Blastocyst implantation depends on the leukemia inhibitory factor (LIF)‐STAT3 pathway.^[^ ^1^ ^]^ We also examined LIF‐STAT3 expression in the LPS‐treated mouse uterus. Compared to the control, LIF immunofluorescence was reduced in the endometrial glands and epithelium of the LPS‐treated group (Figure S5B, Supporting Information). The level of *Lif* mRNA in the LPS‐treated group was also decreased compared with the control group (Figure S5C, Supporting Information). Western blot also showed that the level of p‐STAT3 was also reduced in the LPS‐treated group compared with the control group (Figure S5D, Supporting Information). Western blot analysis and quantification revealed that pyroptosis‐associated proteins were excessively activated by LPS treatment (Figure 7B). Furthermore, LPS also significantly increased the protein levels of mature IL‐18 and IL‐1β in uterine fluid (Figure 7C). GSDMD immunofluorescence in luminal epithelial cells on day 4 of pregnancy was also increased by LPS treatment (Figure 7D). Additionally, a high level of IL‐18 downregulated the protein levels of receptivity‐related proteins (p‐STAT3, HOXA10, and Integrin β3) (Figure 7E). The protein levels of p‐STAT3, HOXA10, and Integrin β3 were also suppressed by high concentrations of IL‐1β (Figure 7F). Either CTSB or LPS was able to induce CTSB expression in cultured epithelial cells (Figure S5E,F, Supporting Information). Those results suggested that LPS induced implantation loss by causing excess pyroptosis.

### 2.8 Pyroptosis in the Mid‐Secretory Endometrium and the RIF Endometrium

**Figure 8:**
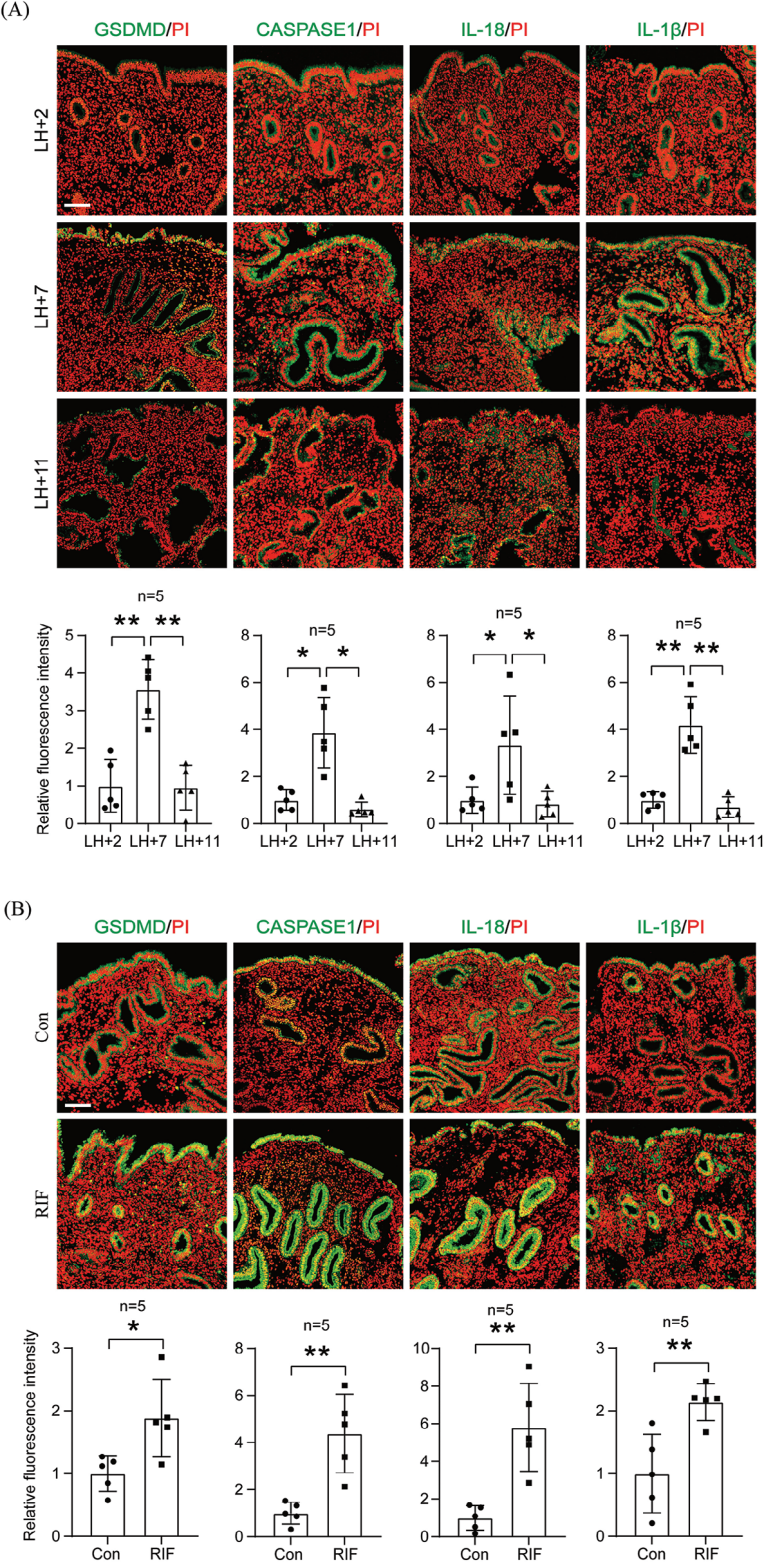
Pyroptosis in mid‐secretory endometrium and RIF endometrium. A) Immunofluorescence and quantification of GSDMD (green), CSASPASE 1 (green), IL‐18 (green), IL‐1β (green), and PI fluorescence (red) in human endometrium on LH+2, LH+7, and LH+11 of the menstrual cycle (*n* = 5 per group). B) Immunofluorescence and quantification of GSDMD (green), CASPASE 1 (green), IL‐18 (green), IL‐1β (green), and PI fluorescence (red) in control and RIF endometrium (*n* = 5 per group). LH+2, early‐secretory phase of the menstrual cycle; LH+7, mid‐secretory phase of the menstrual cycle; LH+11, late‐secretory phase of the menstrual cycle; RIF, recurrent implantation failure. Scale bar = 125 µm. Data were presented as mean ± SD. ^*^: *p* < 0.05; ^**^: *p* < 0.01, by two‐tailed Student's t‐test.

To translate these findings from mice to humans, we examined pyroptosis‐related proteins in the human endometrium. The immunofluorescence levels of GSDMD, CASPASE 1, IL‐1β and IL‐18 during the LH+7 (mid‐secretory phase) of the menstrual cycle were greater than those during both the LH+2 (early‐secretory phase) and LH+11 (late‐secretory phase) of the menstrual cycle (**Figure** **8**A). Recurrent implantation failure (RIF) occurs in 10–15% of IVF couples.^[^ ^3^ ^]^ Compared with those in normal women, the immunofluorescence levels of GSDMD, CASPASE 1, IL‐1β, and IL‐18 were higher in the endometria of RIF patients (Figure 8B). Insulin‐like frowth factor‐binding protein 1(IGFBP1) is a marker of human decidualization.^[^ ^38^ ^]^ Compared with the strong IGFBP1 immunofluorescence in the mid‐secretory endometrium, IGFBP1 immunofluorescence in the endometrium of RIF patients was barely detected (Figure S6A, Supporting Information). We measured CTSB protein levels in the human blastocysts‐conditioned medium by ELISA. Compared to the control medium, the CTSB level was significantly increased in the human blastocysts‐conditioned medium (Figure S6B, Supporting Information). Those results indicate that excessive pyroptosis likely occurs in the human endometria of RIF patients.

## 3 Conclusion and Discussion

Endometrial epithelial cells play a role in mediating the cross‐talk between embryo and endometrium for early successful pregnancy.^[^ ^39^ ^]^ Previous studies have shown that blastocyst‐derived molecules, such as TNF and lactic acid, interact with epithelial cells to mediate implantation and decidualization.^[^ ^10a,c^ ^]^ In this study, we show that blastocyst‐derived CSTB induces pyroptosis in the luminal epithelium at the time of embryo implantation. Pyroptosis‐caused IL‐18 secretion promotes stromal decidualization by activating epithelial ADAM12 and EREG.

The manner of death of uterine luminal epithelial cells is controversial. Previous studies have shown that apoptosis is detected in uterine luminal epithelial cells during embryo attachment.^[^ ^13^ ^]^ However, a recent study indicated that the luminal epithelial cells surrounding the implanting blastocyst are engulfed by trophoblast cells.^[^ ^40^ ^]^ Through different approaches, we showed that CASPASE 1‐mediated pyroptosis naturally occurs in luminal epithelial cells at the time of embryo implantation. Formation of GSDMD pores does not necessarily cause cell death, and such death can be regulated or prevented.^[^ ^41^ ^]^ Accordingly, calcium influx through GSDMD pores initiates a membrane repair program by recruiting the ESCRT machinery to damaged membrane areas.^[^ ^27^ ^]^ Similarly, our data revealed that CHMP4B and CHMP3, members of ESCRT machinery, are present in the luminal epithelium surrounding the implantation site. Based on our results, CTSB‐induced pyroptosis is a rapid and reversible process in endometrial epithelium cells. Because both apoptosis and necrosis are not detected in luminal epithelial at the implantation site, it is possible that pyroptosis in epithelial cells at the implantation site is transient and inflammatory for establishing endometrial receptivity and initiating decidualization, but is not the main mechanism of epithelial cell death.

An appropriate level of inflammation is required for embryo implantation, decidualization, and pregnancy maintenance.^[^ ^42^ ^]^ Abnormal inflammasome activation in the endometrium may adversely affect endometrial receptivity.^[^ ^43^ ^]^ Overactivation of the NLRP3 inflammasome pathway may mediate an abnormal inflammatory response at the maternal‐fetal interface and may be associated with pregnancy complications, such as recurrent implantation failure, pregnancy loss, preterm birth, and preeclampsia.^[^ ^44^ ^]^ In unexplained RIF, endometrial scratching improves endometrial receptivity through the downregulation of NLRP3 and other innate immune‐related molecules.^[^ ^45^ ^]^ IL‐1β and IL‐18, inflammatory markers, are highly expressed in RIF patients.^[^ ^46^ ^]^ Interestingly, in our study, pyroptosis‐related GSDMD, CASPASE 1, and IL‐18 levels are modestly increased in the mid‐secretory phase. Furthermore, the levels of these pyroptosis‐related proteins are significantly increased in RIF endometrium. Our results show that modest pyroptosis is optimal for human endometrium in the mid‐secretory phase. There are different therapeutic options for the clinical treatment of RIF.^[^ ^47^ ^]^ The suppression of overactivated pyroptosis in RIF patients may provide a new choice for treating RIF patients through blocking NLRP3 inflammasome assembly and GSDMD.^[^ ^48^ ^]^ However, further studies are needed to assess the therapeutic methods.

LPS can induce apoptosis and pyroptosis and is used to establish inflammation models.^[^ ^49^ ^]^ In addition, implantation failure can be induced in pregnant mice via LPS.^[^ ^50^ ^]^ Our data suggest that LPS treatment leads to implantation failure by upregulating pyroptosis related protein levels. CTSB, a cysteine proteolytic enzyme found in lysosomes, is induced by LPS through increasing lysosomal membrane permeabilization.^[^ ^51^ ^]^ LPS also stimulates CTSB activity in HK‐2 cells.^[^ ^52^ ^]^ The blastocyst‐derived signal can induce CTSB expression in endometrial lumen epithelium.^[^ ^53^ ^]^ Our results show that both CTSB and LPS induce CTSB expression in mouse endometrial epithelial cells in vitro. Therefore, LPS‐induced abortion may be caused by the excessive expression of CTSB in endometrial epithelium to aggravate pyroptosis. These data indicate that modest pyroptosis is required for embryo implantation and decidualization, but overactivated pyroptosis is harmful.

In our study, there are obvious differences in pyroptosis‐related protein levels between pregnancy and pseudopregnancy on day 4, and between delayed and activated implantation, suggesting that blastocysts are involved in activating pyroptosis. Mouse blastocysts can synthesize and secrete TNF, S100A9, lactic acid, and trypsin during embryo implantation.^[^ ^24^ ^]^ Our results show that only CTSB induces pyroptosis in cultured mouse epithelial cells. CTSB participates in cell death pathways, including apoptosis, ferroptosis, necroptosis, and pyroptosis.^[^ ^54^ ^]^ Previous studies have shown that CTSB is expressed in the uterine epithelium and glands, and trophoblast cells at the implantation site.^[^ ^55^ ^]^ Similarly, our results show that CTSB is located in the uterine epithelium and blastocyst during implantation. In our in vitro study, CTSB is synthesized and secreted in mouse blastocysts, and stimulated by estradiol‐17β. CTSB can trigger the expression of IL‐18 and IL‐1β. It is shown that IL‐1β can increase the attachment of blastocysts to endometrial epithelial cells by improving endometrial receptivity.^[^ ^56^ ^]^ In addition, normal levels of IL‐18 are favorable for uterine receptivity.^[^ ^20a^ ^]^ Our data showed that CTSB trigger epithelial secretion of IL‐1β and IL‐18, which can increase endometrial receptivity. These data indicate that CTSB induces pyroptosis to promote the establishment of endometrial receptivity during embryo implantation. It is shown that CTSB‐deficient mice are fertile and reproduce normally.^[^ ^57^ ^]^ CTSB and cathepsin L are expressed in mouse pregnant uterus.^[^ ^55^, ^58^ ^]^ Both embryo development and decidualization are inhibited by E‐64, a synthetic inhibitor of cathepsins B and L.^[^ ^57^ ^]^ It is possible to have a redundance mechanism for CTSB during mouse early pregnancy.

Our data suggest that only IL‐18 stimulates endometrial decidualization through epithelial‐stromal cross‐talk. EREG, AREG, and HB‐EGF act as local mediators for uterine preparation and embryo‐uterine interactions during implantation.^[^ ^59^ ^]^ EGF family members are shed by ADAMs to release the EGF domain to activate the downstream signaling pathway.^[^ ^60^ ^]^ EREG is expressed exclusively in the luminal epithelium and underlying stroma surrounding the implanting blastocyst on day 4.5 and day 5.^[^ ^33^ ^]^ Meanwhile, EREG is involved in stress, inflammation, wound healing, angiogenesis, proliferation, migration, differentiation, and regeneration.^[^ ^60^, ^61^ ^]^ It is shown that EREG expression is stimulated by IL‐1β, IL‐6, and IL‐17A.^[^ ^34^ ^]^ In our study, IL‐18, but not IL‐1β, promotes mouse decidualization through stimulating EREG when uterine epithelial cells were cocultured with stromal cells. In vitro, decidualization in humans is significantly enhanced by EREG.^[^ ^62^ ^]^ In our study, EREG is cleaved by ADAM12. ADAM12 is expressed in the luminal and glandular epithelium of the human endometrium.^[^ ^35^ ^]^ ADAM12 knockdown suppresses decidualization.^[^ ^63^ ^]^ Our results show that IL‐18 increases the expression of ADAM12 and EREG in endometrial epithelial cells. Our results further indicate that IL‐18 promotes *Prl8a2*, *Prl3c1*, and *E2f8* expression in the co‐culture of epithelial cells and stromal cells. Therefore, we conclude that IL‐18 stimulates endometrial decidualization through epithelial‐stromal cross‐talk.

In conclusion, we found that blastocyst‐derived CTSB facilitates embryo implantation by inducting pyroptosis in the uterine epithelium and promoting decidualization through activating epithelial ADAM12 and EREG. Moreover, proper pyroptosis is critical for embryo implantation and decidualization. Overactivated pyroptosis may lead to pregnancy failure.

## 4 Experimental Section

### Animals and Treatments

Mature CD1 mice (6–8 weeks old) were housed in a controlled environment (12 h light). All of the mouse treatments were approved by the Institutional Animal Care and Use Committee of South China Agricultural University (No. 2021f085). Female mice were mated with fertile or vasectomized males to obtain pregnant or pseudopregnant mice, respectively. The day of the vaginal plug was defined as day 1 of pregnancy or pseudopregnancy. Pregnancy from days 1 to 4 was confirmed by flushing embryos from the oviducts or uteri. Pregnancy on day 5 was confirmed by tail intravenous injection of 0.1 ml of 1% Chicago blue dye (C8679, Sigma–Aldrich, St. Louis, MO). The uteri on different days of pregnancy were collected for further studies.

### Collection of Human Endometrium during Menstrual Cycle and from RIF Patients

This study was approved by the Ethics Committee of the Drum Tower Hospital. All women provided written informed consent. Endometrial biopsy samples were collected from women who underwent assisted reproductive technology (ART) treatment at the Center for Reproductive Medicine, Nanjing Drum Tower Hospital.

The control group consisted of patients who underwent assisted reproductive treatment specifically for male infertility and successfully conceived following the subsequent transplantation. The Repeated Implant Failure (RIF) group consisted of patients who had undergone 3 or more successive embryo transfers, with the transfer of more than 4 high‐quality cleavage stage embryos or more than 2 high‐quality blastocysts, but without successful implantation. Each group had five samples.

Ovulation was observed beginning on the 12th day of menstruation, and HCG 5000 IU was given when the leading follicle reached 18 mm diameter, which was designated as LH+0. Endometrial biopsies were obtained on LH+2, LH+7, and LH+11 from patients who did not undergone embryo transfer following IVF treatment due to the possibility of OHSS or fertilization failure in stimulated cycles. Each group had five samples.

The participants in the study were women between the ages of 25 and 40 who had a healthy body mass index (BMI) ranging from 18 to 25 kg m^−2^. They also had regular menstrual cycles lasting between 26 and 35 days and were evaluated by a medical professional to ensure they did not have any hormonal abnormalities. Uterine abnormalities, adenomyosis, endometriosis, uterine fibroids, polycystic ovary syndrome (PCOS), and hydrosalpinx were ruled out.

### Intrauterine Injection

As previously described,^[^ ^64^ ^]^ at 9:00 in the morning of day 4 of pregnancy, female mice were intraluminally injected with 4 µL of 1 µm disulfiram (pyroptosis inhibitor, PHR1690, Sigma–Aldrich), 1 µm CA‐074 Me (CTSB inhibitor, S7420, Selleck, Shanghai, China), 1 µm NLRP3‐IN‐21 (NLRP3 inhibitor, HY‐149604, MedChemExpress, NJ, USA) or 100 ng mL^−1^ IL‐18 binding protein (for inhibiting IL‐18, 50206‐M08H, Sino Biological, Beijing, China) into one uterine horn using a 26‐gauge Hamilton syringe. Mice were sacrificed on day 5 of pregnancy to collect implantation sites by tail intravenous injection 0.1 ml of 1% Chicago blue dye.

At 9:00 in the morning on day 4 of pseudopregnancy, 6 mice were intraluminally injected with 4 µL of mouse CTSB protein (10 ng/mL, 50084‐M08H, Sino Biological) into one uterine horn using a 26‐gauge Hamilton syringe. After mice were sacrificed 1 h after injection, the uteri were collected for further analysis.

### Delayed Implantation and Activation

As previously described,^[^ ^65^ ^]^ At 8:30–9:00 in the morning on day 4 of pregnancy, 12 female mice were ovariectomized and daily injected with progesterone (1 mg/mouse, E1024, Sigma–Aldrich) to maintain delayed implantation from days 5 to 7. 6 mice under delayed implantation were activated by injecting 25 ng estradiol‐17β (E8875, Sigma–Aldrich) in oil per mouse on day 7 of pregnancy.

The dormant and reactivated blastocysts were collected as previously described.^[^ ^24^ ^]^ In brief, reactivated blastocysts were flushed from uteri with M2 medium (M7167, Sigma–Aldrich) 12 h after delayed implantation was terminated by estradiol‐17β. Dormant blastocysts were flushed from uteri 12 h after mice under delayed implantation were injected with progesterone on day 7 of pregnancy. Dormant blastocysts were cultured in KSOM (MR‐106‐D, Merck, Darmstadt, Germany) with different concentrations of estradiol‐17β for 12 h.

### LPS‐Induced Abortion

Abortion was induced as previously described.^[^ ^36^ ^]^ Briefly, 12 female mice on day 3 of pregnancy were intraperitoneally injected with LPS (50 µg/mouse, L4391, Sigma–Aldrich) or saline. When mice were sacrificed on day 4 or 5 of pregnancy, the uteri were collected for further studies.

### Preparation and Uterine Transfer of Blastocyst‐Size Beads

As previously described,^[^ ^10a^ ^]^ Affi‐Gel beads (1,537,302, Bio‐Rad, Hercules, CA, USA) of the blastocyst‐size were incubated with IL‐18 (10 ng mL^−1^, 50073‐MNCE, SinoBiological), IL‐1β (10 ng mL^−1^, 211‐11B, Peprotech, Rocky Hill, USA) or CTSB (10 ng mL^−1^, 211‐11B, SinoBiological) in PBS at 37 °C for 4 h. BSA was served as control. After being washed three times in PBS, 15 beads were transferred into the uterine horn of day 4 pseudopregnant mice. The blue bands for attachment reaction were observed by intravenous injection of Chicago blue 24 h after transfer.

### Collection and Treatment of Embryos

Embryo collection and culture were performed as previously described.^[^ ^66^ ^]^ Blastocysts collected from day 4 pregnant mice were cultured in KSOM (Merck, MR‐106‐D, Darmstadt, Germany) for 6 h in a 5% CO_2_ incubator at 37 °C. The cultured medium was collected for further studies.

Reactivated blastocysts were collected from uteri 12 h after delayed implantation was terminated by estradiol‐17β. Dormant blastocysts were flushed from uteri 12 h after mice under delayed implantation were injected with progesterone on day 7 of pregnancy. Dormant blastocysts were cultured in KSOM with different concentrations of estradiol‐17β for 12 h. Embryos and the cultured medium were collected for further studies.

### Enzyme‐Linked Immunosorbent Assay

Uterine luminal fluid was collected by flushing the uterine lumen with 100 µL of saline on day 4 of pregnancy or pseudopregnancy and centrifuged at 1000 xg for 5 min to remove the cellular debris and embryos. Levels of IL‐18 and IL‐1β in uterine luminal fluid or cultured medium were measured using commercial enzyme‐linked immunosorbent assay kits (E‐EL‐M0730c, Elabscience, Wuhan, China; KE10003, Proteintech, Chicago, USA) according to the manufacturer's instructions. Levels of S100A8/S100A9 (EM67RB, ThermoFisher, Waltham, MA, USA) and LDH (JL13877, Jianglai organism, Shanghai, China) in culture medium were measured according to the manufacturer's instructions after epithelial cells were treated with CTSB for 5 min. After blastocysts were collected from day 4 pregnant mice were cultured in KSOM (20 µL) for 6 h in a 5% CO_2_ incubator at 37 °C, CTSB level in the cultured medium was measured as per the instruction (E‐EL‐H6151, Elabscience).

### Isolation and Treatment of Mouse Endometrial Epithelial Cells

Mouse endometrial epithelial cells were isolated as previously described.^[^ ^10a^ ^]^ Uteri on day 4 of pseudopregnancy were split longitudinally, washed with Hanks’ balanced salt solution (HBSS, H4891, Sigma–Aldrich), and digested with 0.3% trypsin (0458, Amresco, Cleveland, USA) and 6 mg mL^−1^ dispase II (049,420,7801, Roche Applied Science, Basel, Switzerland) in HBSS for 1.5 h at 4 °C, 30 min at room temperature and 10 min at 37 °C. After washing in HBSS, the luminal epithelial cells were collected and cultured in DMEM/F12 (D2906, Sigma–Aldrich) containing 10% charcoal‐treated FBS (040011A, Biological Industries, Cromwell, CT, USA). Cultured epithelial cells were treated with 5, 50 and 500 ng mL^−1^ mouse S100A9 (50284‐M07E, Sino Biological), 1, 10 and 100 ng mL^−1^ mouse TNF (410‐MT‐010, Bio‐Techne, Minnesota, USA), 10 ng mL^−1^ mouse IL‐18 (50073‐MNCE, Sino Biological), 100 ng mL^−1^ mouse IL‐18BP (50206‐M08H, Sino Biological), 10 ng mL^−1^ mouse IL‐1β (211‐11B, Peprotech), or 10 ng mL^−1^ mouse CTSB (50084‐M08H, Sino Biological) for 3 h. Cultured endometrial epithelial cells were also treated with mouse CTSB and disulfiram (pyroptosis inhibitor, PHR1690, Sigma–Aldrich), CTSB and CA‐074 Me (CTSB inhibitor, S7420, Selleck, Shanghai, China), CTSB and NLRP3‐IN‐21, disulfiram, NLRP3‐IN‐21 or CA‐074 Me for 3 h. Endometrial epithelial cells were treated with CTSB and PI, EthD‐III (HY‐D1723, MedChemExpress) or DRAQ7 (D15106, ThermoFisher, Waltham, MA, USA) for 0, 3, 5, 8, and 10 min. A fluorescence signal was detected.

### Isolation and Treatment of Mouse Endometrial Stromal Cells

Mouse endometrial stromal cells were isolated as previously described.^[^ ^10a^ ^]^ Mouse uteri on day 4 of pseudopregnancy were split longitudinally, washed with HBSS, and digested with 6 mg mL^−1^ dispase II and 1% trypsin in HBSS for 1 h at 4 °C, 1 h at room temperature, 10 min at 37 °C. After washing in HBSS, the luminal epithelial cells were removed by centrifugation. The remaining uteri were incubated with 0.15 mg mL^−1^ collagenase I (17100‐017, Invitrogen, Houston, TX, USA) in HBSS for 35 min at 37 °C. Endometrial stromal cells were collected and seeded in culture plates with DMEM/F12 containing 10% FBS. The stromal cells were treated with estradiol‐17β (10 nm) and progesterone (1 µm) to induce the in vitro decidualization. Cultured stromal cells were treated with mouse EREG (1608EP050, Bio‐Techne, Minnesota, USA) for 24 h.

### Isolation and Culture of Mouse Endometrial Organoids

Mouse endometrial organoids were prepared as previously described.^[^ ^10a^ ^]^ Mouse uteri on day 4 of pseudopregnancy were split longitudinally, washed with HBSS, and digested with 6 mg mL^−1^ dispase II and 1% trypsin in HBSS for 1 h at 4 °C, 1 h at 25 °C, and 10 min at 37 °C. After washing in HBSS, the epithelial cells were collected and resuspended in a mixture of 70% ice‐precooled ECM (356,231, BD biocoat, Becton‐Dickinson, MA) and 30% DMEM/F12. The suspended cells were seeded into the preheated 24‐well plates in drops and cultured in DMEM/F12 organoid medium containing 1% ITS‐G (PB180429, Procell, Wuhan, China), 2 mM L‐glutamine (49,419, Sigma–Aldrich), 1 mm nicotinamide (49,419, Sigma–Aldrich), 2% B27 (17504‐044, Gibco, Grand Island, NY), 1% N2 (17502–048, Gibco), 50 ng mL^−1^ EGF (HY‐P7067, MedChemExpress, NJ, USA),100 ng mL^−1^ FGF‐basic (HY‐P7066, MedChemExpress), 100 ng mL^−1^ Noggin (HY‐P70785, MedChemExpress), 200 ng mL^−1^ WNT‐3A (315–20, Peprotech, Rocky Hill, USA), 200 ng mL^−1^ R‐Spondin‐1 (HY‐P76012, MedChemExpress), and 0.5 µm A83‐01 (HY‐10432, MedChemExpress). Cultured endometrial organoids were treated with mouse CTSB (50084‐M08H, Sino Biological), CTSB and disulfiram (pyroptosis inhibitor, PHR1690, Sigma–Aldrich), CTSB and CA‐074 Me (CTSB inhibitor, S7420, Selleck, Shanghai, China), CTSB and NLRP3‐IN‐21, disulfiram, NLRP3‐IN‐21 or CA‐074 Me for 3 h.

### Co‐Culture of Endometrial Epithelial with Stromal Cells

As previously described,^[^ ^10a^ ^]^ uterine epithelial cells were seeded onto the glass slipper, and stromal cells were seeded in 24‐well plates. After the density of epithelial and stromal cells reached a proper level, the glass slipper containing cultured epithelial cells was placed into 24‐well plates containing cultured stromal cells. The glass slippers were supported by four sterile plastic pillars. The cocultured epithelial and stromal cells were treated with IL‐18, IL‐18BP, or CTSB.

### Embryo Adhesion Assay

The embryo adhesion assay was conducted as previously described.^[^ ^67^ ^]^ Briefly, blastocysts collected from day 4 pregnant mice were transferred onto cultured epithelial cells in 24‐well plates and co‐cultured with mouse IL‐18 or IL‐1β for 72 h. The numbers of the adhered blastocysts were counted to calculate the adhesion rates. The adhesion rate was calculated as the number of adhered embryos/the number of total embryos X 100%.

### Plasmid Transfection

Mouse endometrial epithelial cells were transfected with pBOB‐mGSDMD‐NT‐Flag plasmid using a Lipofectamine 3000 kit (L3000015, ThermoFisher, Waltham, MA, USA) for 24 h. The pBOB‐mGSDMD‐NT‐Flag plasmid was kindly provided by Professor Jiahui Han of Xiamen University, China. The transfected cells were treated with CTSB for subsequent analysis.

### RNA Interference

The siRNAs were purchased from the Ribobio Co.Ltd. (Guangzhou, China). The sequence of mouse siADAM12 was GAAAGTTAAGCAGCGATTA. Mouse endometrial epithelial cells were transfected with ADAM12 siRNA according to the kit Lipofectamine 2000 kit (11,668,019, Invitrogen, Carlsbad, CA) for 12 h. The transfected cells were used for subsequent analysis.

### Western Blot

Western blot was performed as previously described.^[^ ^62^ ^]^ Briefly, tissues or cultured cells were lysed on ice in lysis buffer (50 mm Tris‐HCl, pH 7.5; 150 mM NaCl; 0.25% sodium deoxycholate and 1% Triton X‐100). The protein concentrations were quantified by the BCA method (ThermoFisher Scientific, Waltham, MA). The Protein samples were separated on 10% SDS‐polyacrylamide gel electrophoresis (SDS/PAGE) and transferred onto polyvinylidene fluoride (PVDF) membranes (IPVH00010, Millipore, Billerica, MA). The membranes were blocked with 5% non‐fat milk (BBI Life Sciences, Shanghai, China) for 1 h, and incubated with each primary antibody overnight at 4 °C, including anti‐GAPDH (1:1000, SC‐32233, Santa Cruz Biotechnology, Dallas, TX), anti‐NLRP3 (1:1000, NBP2‐12446, NOVUS, Colorado, USA), anti‐CASPASE 1 (1:1000, ab179515, Abcam, Cambridge, UK), anti‐GSDMD (1:1000, ab219800, Abcam), anti‐IL‐18 (1:1000, ab207323, Abcam), anti‐IL‐1β (1:1000, ab234437, Abcam), anti‐CTSB (1:1000, ab214428, Abcam), anti‐STAT3 (1:1000, 9139s, Cell Signaling Technology, Danvers, MA), anti‐p‐STAT3 (1:1000, 9131s, Cell Signaling Technology), anti‐EREG (1:1000, ab233512, Abcam), anti‐AREG (1:1000, SC‐74501, Santa Cruz), anti‐HB‐EGF (1:1000, SC28908, Santa Cruz), anti‐HOXA10 (1:1000, Sc‐28602, Santa Cruz), anti‐integrin β3 (1:1000, 13166s, Cell Signaling Technology), anti‐ADAM12 (1:1000, A7940, Abclonal, Wuhan, China), anti‐ASC (1:200, ab219800, Abcam), anti‐P‐RIP3 (1:200, 91702S, Cell Signaling Technology), anti‐P‐MLKL (1:200, 37333S, Cell Signaling Technology), anti‐cleaved CASPASE 3 (1:200, 9661s, Cell Signaling Technology), anti‐CASPASE 11 (1:200, ab180673, Abcam). After the membranes were incubated with horseradish peroxidase (HRP)‐conjugated secondary antibody (1:5000, Invitrogen) for 1 h at 25 °C, the signals were visualized with ECL chemiluminescent kit (Millipore, USA) by employing 5200 Tanon Imaging System.

### Immunofluorescence

Immunofluorescence was performed as previously described.^[^ ^10c^ ^]^ Uteri were fixed in 10% neutral buffered formalin and paraffin‐embedded. Paraffin sections were deparaffinized, rehydrated, and antigen‐retrieved with Tris/EDTA buffer (pH 9.0) for 10 min by microwaving. Sections were blocked with 10% horse serum for 1 h at 37 °C and incubated with each primary antibody overnight at 4 °C, including anti‐DYKDDDDK tag (1:200, 20543‐1‐AP, Proteintech, Chicago, USA), anti‐CASPASE 1 (1:200, ab179515, Abcam), anti‐NLRP3 (1:200, NBP2‐12446, NOVUS, Colorado, USA), anti‐GSDMD (1:1000, ab219800, Abcam), anti‐IL‐18 (1:1000, ab207323, Abcam), anti‐GSDMD‐N (1:200, ER1901‐37, CUSABIO, Wuhan, China), anti‐EREG (1:200, ab233512, Abcam), anti‐ADAM12 (1:200, ab276047, Abcam), anti‐CHMP4B (1:200, CSB‐PA078194 CUSABIO), anti‐CHMP3 (1:200, K108653P, Solarbio, Beijing, China), anti‐LIF (1:200, ab113262, Abcam), anti‐IGFBP1 (1:200, SC‐55474, Santa Cruz), anti‐COX2 (1:200, 12282T, Cell Signaling Technology), anti‐WNT4 (1:200, sc‐376279, Santa Cruz), anti‐BMP2 (1:200, NBP1‐19751, NOVUS), anti‐Hand2 (1:200, sc‐9409, Santa Cruz), anti‐CTSB (1:1000, ab214428, Abcam), anti‐P‐RIP3 (1:200, 91702S, Cell Signaling Technology), anti‐P‐MLKL (1:200, 37333S, Cell Signaling Technology), anti‐NINJ1 (1:200, ab213695, Abcam) in a humid chamber. Normal rabbit IgG (2729, Cell Signaling Technology) was used as the negative control. After washing three times in PBS, sections were incubated with Alexa 488‐conjugated second antibody (Jackson ImmunoResearch Laboratories, West Grove, PA) for 30 min at 37 °C and counterstained with propidium iodide (PI, Sigma–Aldrich). The fluorescence signals were acquired by Leica TCS SP8 scanning laser confocal microscope and Nikon C2 confocal microscope.

Immunofluorescence of mouse embryos was performed as previously described.^[^ ^24^ ^]^ In brief, embryos were fixed with 4% paraformaldehyde (PFA, Sigma–Aldrich) in PBS for 30 min and permeabilized with 2.5% Tween 20 for 5 min. After washing three times in 0.1% BSA in PBS, embryos were incubated with anti‐CTSB antibody (1:200, ab214428H, Abcam) overnights at 4 °C in a humid chamber. After washing three times with 0.5% Triton X‐100 and 0.1% BSA in PBS, embryos were incubated with Alexa 488‐conjugated second antibody (Jackson ImmunoResearch Laboratories, West Grove, PA) and counterstained with DAPI (Sigma–Aldrich) for 30 min at 37 °C. The fluorescence signals were acquired by Leica TCS SP8 scanning laser confocal microscope and Nikon C2 confocal microscope.

### Quantitative Polymerase Chain Reaction

qPCR was performed as previously described.^[^ ^68^ ^]^ Total RNAs were extracted by using TRIzol reagent (9109, Takara, Kusatsu, Japan). cDNA was obtained by reverse‐transcription of HiScript II Q RT SuperMix (R222‐01‐AB, Vazyme, Nanjing, China) by using BIO‐RAD T100. qPCR was performed with a SYBR Premix Ex Taq kit (Q311‐02‐AA, Vazyme) on the qTOWER3G (Analytik Jena, Germany). The data were analyzed by the 2^‐△△^ CT method and normalized to RPL7. Primer sequences were provided in the Supporting Information.

### Statistical Analysis

Statistical analyses were conducted in GraphPad Prism 9 software. The data were expressed as the mean ± standard deviation (SD) unless otherwise specified. Two‐tailed Student's t‐test was used to compare the two groups. A one‐way ANOVA test was used to compare more than two groups. The signal intensity for Western blot and immunofluorescence were carried out using the ImageJ software. The level of each band for Western blot was normalized to the level of GAPDH. The signal intensity for immunofluorescence was normalized to the mean of the control group. The sample size (n) was shown in the corresponding figure legends. Statistical significance was defined as ^*^: *p* < 0.05; ^**^: *p* < 0.01; ^***^: *p* < 0.001, ns: not significant.

## Conflict of Interest

The authors declare no conflict of interest.

## Author Contributions

M.‐Y.L. designed and conducted the study, and wrote and revised the paper. Y.W., H.‐L.T., Y.W., B.L., and Y.‐Y.H. provided technical and material support. All authors approved the final manuscript.

## Supporting information



Supporting Information

## Data Availability

The data that support the findings of this study are available from the corresponding author upon reasonable request.

## References

[1] H. Wang , S. K. Dey , Nat. Rev. Genet. 2006, 7, 185. 16485018 10.1038/nrg1808

[2] E. R. Norwitz , D. J. Schust , S. J. Fisher , N. Engl. J. Med. 2001, 345, 1400. 11794174 10.1056/NEJMra000763

[3] R. Pourakbari , H. Ahmadi , M. Yousefi , L. Aghebati‐Maleki , Life Sci. 2020, 258, 118181. 32763291 10.1016/j.lfs.2020.118181

[4] A. Simon , N. Laufer , Fertil. Steril. 2012, 97, 1039. 22464086 10.1016/j.fertnstert.2012.03.010

[5] M. Namlı Kalem , N. Akgun , Z. Kalem , B. Bakirarar , T. Celik , J. Assist. Reprod. Genet. 2017, 34, 1501. 28707148 10.1007/s10815-017-0992-5 PMC5700001

[6] S. Li , J. Wang , Y. Cheng , D. Zhou , T. Yin , W. Xu , N. Yu , J. Yang , J. Reprod. Immunol. 2017, 119, 15. 27915038 10.1016/j.jri.2016.11.006

[7] Y. Zhao , T. Zhang , X. Guo , C. K. Wong , X. Chen , Y. L. Chan , C. C. Wang , S. Laird , T. C. Li , Fertil. Steril. 2021, 115, 1044. 33272613 10.1016/j.fertnstert.2020.10.031

[8] Y. Gnainsky , I. Granot , P. B. Aldo , A. Barash , Y. Or , E. Schechtman , G. Mor , N. Dekel , Fertil. Steril. 2010, 94, 2030. 20338560 10.1016/j.fertnstert.2010.02.022 PMC3025806

[9] D. K. Li , L. Liu , R. Odouli , BMJ 2003, 327, 368. 12919986 10.1136/bmj.327.7411.368 PMC175811

[10] a) S. T. Chen , W. W. Shi , Y. Q. Lin , Z. S. Yang , Y. Wang , M. Y. Li , Y. Li , A. X. Liu , Y. Hu , Z. M. Yang , Elife 2023, 12, e82970; 37458359 10.7554/eLife.82970 PMC10374279

[11] a) I. Granot , Y. Gnainsky , N. Dekel , Reproduction 2012, 144, 661; 23028125 10.1530/REP-12-0217

[12] S. Christgen , R. E. Tweedell , T. D. Kanneganti , Pharmacol. Ther. 2022, 232, 108010. 34619283 10.1016/j.pharmthera.2021.108010 PMC8930427

[13] S. Pampfer , I. Donnay , Cell Death Differ. 1999, 6, 533. 10381643 10.1038/sj.cdd.4400516

[14] P. Broz , P. Pelegrín , F. Shao , Nat. Rev. Immunol. 2020, 20, 143. 31690840 10.1038/s41577-019-0228-2

[15] J. Shi , W. Gao , F. Shao , Trends Biochem. Sci. 2017, 42, 245. 27932073 10.1016/j.tibs.2016.10.004

[16] T. Bergsbaken , S. L. Fink , B. T. Cookson , Nat. Rev. Microbiol. 2009, 7, 99. 19148178 10.1038/nrmicro2070 PMC2910423

[17] Y. Xue , D. E. Tuipulotu , W. H. Tan , C. Kay , S. M. Man , Trends Immunol. 2019, 40, 1035. 31662274 10.1016/j.it.2019.09.005

[18] L. Sborgi , S. Ruhl , E. Mulvihill , J. Pipercevic , R. Heilig , H. Stahlberg , C. J. Farady , D. J. Muller , P. Broz , S. Hiller , EMBO J. 2016, 35, 1766. 27418190 10.15252/embj.201694696 PMC5010048

[19] X. Wei , F. Xie , X. Zhou , Y. Wu , H. Yan , T. Liu , J. Huang , F. Wang , F. Zhou , L. Zhang , Cell Mol. Immunol. 2022, 19, 971. 35970871 10.1038/s41423-022-00905-x PMC9376585

[20] a) N. Lédée‐Bataille , K. Bonnet‐Chea , G. Hosny , S. Dubanchet , R. Frydman , G. Chaouat , Fertil. Steril. 2005, 83, 598; 15749487 10.1016/j.fertnstert.2004.11.021

[21] N. Winsor , C. Krustev , J. Bruce , D. J. Philpott , S. E. Girardin , Cell. Microbiol. 2019, 21, e13079. 31265745 10.1111/cmi.13079

[22] R. K. S. Malireddi , S. Kesavardhana , T. D. Kanneganti , Front. Cell Infect. Microbiol. 2019, 9, 406. 31850239 10.3389/fcimb.2019.00406 PMC6902032

[23] W. T. Yan , W. J. Zhao , X. M. Hu , X. X. Ban , W. Y. Ning , H. Wan , Q. Zhang , K. Xiong , Neural Regen. Res. 2023, 18, 485. 35900430 10.4103/1673-5374.346545 PMC9396479

[24] B. He , H. Zhang , J. Wang , M. Liu , Y. Sun , C. Guo , J. Lu , H. Wang , S. Kong , Proc. Natl. Acad. Sci. 2019, 116, 16621. 31346081 10.1073/pnas.1900401116 PMC6697802

[25] C. Liu , Q. Yao , T. Hu , Z. Cai , Q. Xie , J. Zhao , Y. Yuan , J. Ni , Q. Q. Wu , Mol. Ther. Nucleic Acids 2022, 30, 198. 36250207 10.1016/j.omtn.2022.09.019 PMC9554743

[26] a) N. Kayagaki , O. S. Kornfeld , B. L. Lee , I. B. Stowe , K. O'Rourke , Q. Li , W. Sandoval , D. Yan , J. Kang , M. Xu , J. Zhang , W. P. Lee , B. S. McKenzie , G. Ulas , J. Payandeh , M. Roose‐Girma , Z. Modrusan , R. Reja , M. Sagolla , J. D. Webster , V. Cho , T. D. Andrews , L. X. Morris , L. A. Miosge , C. C. Goodnow , E. M. Bertram , V. M. Dixit , Nature 2021, 591, 131; 33472215 10.1038/s41586-021-03218-7

[27] S. Rühl , K. Shkarina , B. Demarco , R. Heilig , J. C. Santos , P. Broz , Science 2018, 362, 956. 30467171 10.1126/science.aar7607

[28] a) M. Pruenster , R. Immler , J. Roth , T. Kuchler , T. Bromberger , M. Napoli , K. Nussbaumer , I. Rohwedder , L. M. Wackerbarth , C. Piantoni , K. Hennis , D. Fink , S. Kallabis , T. Schroll , S. Masgrau‐Alsina , A. Budke , W. Liu , D. Vestweber , C. Wahl‐Schott , J. Roth , F. Meissner , M. Moser , T. Vogl , V. Hornung , P. Broz , M. Sperandio , Nat. Immunol. 2023, 24, 2021; 37903858 10.1038/s41590-023-01656-1 PMC10681899

[29] J. J. Hu , X. Liu , S. Xia , Z. Zhang , Y. Zhang , J. Zhao , J. Ruan , X. Luo , X. Lou , Y. Bai , J. Wang , L. R. Hollingsworth , V. G. Magupalli , L. Zhao , H. R. Luo , J. Kim , J. Lieberman , H. Wu , Nat. Immunol. 2020, 21, 736. 32367036 10.1038/s41590-020-0669-6 PMC7316630

[30] W. P. Arend , G. Palmer , C. Gabay , Immunol. Rev. 2008, 223, 20. 18613828 10.1111/j.1600-065X.2008.00624.x

[31] X. Cai , Y. Jiang , Z. Cao , M. Zhang , N. Kong , L. Yu , Y. Tang , S. Kong , W. Deng , H. Wang , J. Sun , L. Ding , R. Jiang , H. Sun , G. Yan , EBio Med. 2023, 88, 104433. 10.1016/j.ebiom.2022.104433 PMC9841229 36623453

[32] a) C. A. Rasmussen , K. E. Orwig , S. Vellucci , M. J. Soares , Biol. Reprod. 1997, 56, 647; 9047009 10.1095/biolreprod56.3.647

[33] S. K. Das , N. Das , J. Wang , H. Lim , B. Schryver , G. D. Plowman , S. K. Dey , Dev. Biol. 1997, 190, 178. 9344537 10.1006/dbio.1997.8694

[34] a) Y. Kyotani , A. Itaya‐Hironaka , A. Yamauchi , S. Sakuramoto‐Tsuchida , M. Makino , S. Takasawa , M. Yoshizumi , FEBS Open Bio 2018, 8, 868; 10.1002/2211-5463.12430 PMC5929938 29744301

[35] M. Kveiborg , R. Albrechtsen , J. R. Couchman , U. M. Wewer , Int. J. Biochem. Cell Biol. 2008, 40, 1685. 18342566 10.1016/j.biocel.2008.01.025

[36] a) X. H. Zhong , W. Y. Shi , A. T. Ma , X. C. Gong , X. H. Zhai , T. Zhang , X. D. Wang , Am. J. Chin. Med. 2008, 36, 141; 18306457 10.1142/S0192415X08005655

[37] a) J. Y. Kang , M. M. Xu , Y. Sun , Z. X. Ding , Y. Y. Wei , D. W. Zhang , Y. G. Wang , J. L. Shen , H. M. Wu , G. H. Fei , Int. Immunopharmacol. 2022, 109, 108782; 35468366 10.1016/j.intimp.2022.108782

[38] M. Al‐Sabbagh , L. Fusi , J. Higham , Y. Lee , K. Lei , A. C. Hanyaloglu , E. W. Lam , M. Christian , J. J. Brosens , Endocrinology 2011, 152, 730. 21159852 10.1210/en.2010-0899 PMC3037160

[39] X. Ye , Trends Endocrinol. Metab. 2020, 31, 165. 31866217 10.1016/j.tem.2019.11.008 PMC6983336

[40] Y. Li , X. Sun , S. K. Dey , Cell Rep. 2015, 11, 358. 25865893 10.1016/j.celrep.2015.03.035 PMC5089169

[41] J. Lieberman , H. Wu , J. C. Kagan , Sci. Immunol. 2019, 4, eaav1447. 31492708 10.1126/sciimmunol.aav1447 PMC7004224

[42] M. Nadeau‐Vallée , D. Obari , J. Palacios , M. Brien , C. Duval , S. Chemtob , S. Girard , Reproduction 2016, 152, R277. 27679863 10.1530/REP-16-0453

[43] J. Sehring , A. Beltsos , R. Jeelani , Placenta 2022, 117, 179. 34929458 10.1016/j.placenta.2021.12.015

[44] C. N. Balci , N. Acar , J. Reprod. Immunol. 2024, 161, 104173. 38043434 10.1016/j.jri.2023.104173

[45] S. Aghajanpour , E. Hosseini , E. Amirchaghmaghi , Z. Zandieh , F. Amjadi , A. Yahyaei , Z. Zolfaghari , K. Aflatoonian , M. Ashrafi , R. Aflatoonian , J. Reprod. Immunol. 2021, 148, 103426. 34653814 10.1016/j.jri.2021.103426

[46] V. Cela , S. Daniele , M. E. R. Obino , M. Ruggiero , E. Zappelli , L. Ceccarelli , F. Papini , I. Marzi , G. Scarfò , F. Tosi , F. Franzoni , C. Martini , P. G. Artini , J. Clin. Med. 2022, 11, 2481. 35566605 10.3390/jcm11092481 PMC9101226

[47] J. Ma , W. Gao , D. Li , Front. Endocrinol. 2022, 13, 1061766. 10.3389/fendo.2022.1061766 PMC9849692 36686483

[48] R. C. Coll , K. Schroder , P. Pelegrín , Trends Pharmacol. Sci. 2022, 43, 653. 35513901 10.1016/j.tips.2022.04.003

[49] a) P. Shen , S. Ji , X. Li , Q. Yang , B. Xu , C. K. C. Wong , L. Wang , L. Li , Front. Endocrinol. 2022, 13, 886085; 10.3389/fendo.2022.886085 PMC9259990 35813649

[50] a) K. Deb , M. M. Chaturvedi , Y. K. Jaiswal , Infect. Dis. Obstet. Gynecol. 2005, 13, 125; 16126496 10.1080/10647440500147885 PMC1784569

[51] N. Chen , Z. Ou , W. Zhang , X. Zhu , P. Li , J. Gong , Cell Prolif. 2018, 51, e12487. 30084208 10.1111/cpr.12487 PMC6528937

[52] Y. Wang , W. Xi , X. Zhang , X. Bi , B. Liu , X. Zheng , X. Chi , Front. Immunol. 2022, 13, 1053754. 36713420 10.3389/fimmu.2022.1053754 PMC9880165

[53] P. Wang , S. Du , C. Guo , Z. Ni , Z. Huang , N. Deng , H. Bao , W. Deng , J. Lu , S. Kong , H. Zhang , H. Wang , Autophagy 2024, 20, 58. 37584546 10.1080/15548627.2023.2247747 PMC10761037

[54] Z. Xie , M. Zhao , C. Yan , W. Kong , F. Lan , Narengaowa , S. Zhao , Q. Yang , Z. Bai , H. Qing , J. Ni , Cell Death Dis. 2023, 14, 255. 37031185 10.1038/s41419-023-05786-0 PMC10082344

[55] S. Afonso , L. Romagnano , B. Babiarz , Development 1997, 124, 3415. 9310336 10.1242/dev.124.17.3415

[56] A. Bourdiec , A. Akoum , Med. Sci. 2014, 30, 644. 10.1051/medsci/20143006014 25014455

[57] W. Halangk , M. M. Lerch , B. Brandt‐Nedelev , W. Roth , M. Ruthenbuerger , T. Reinheckel , W. Domschke , H. Lippert , C. Peters , J. Deussing , J. Clin. Invest. 2000, 106, 773. 10995788 10.1172/JCI9411 PMC381392

[58] J. E. Kim , M. Y. Lee , M. J. Kang , J. Byun , J. B. Jo , H. Y. Yang , J. H. Kim , K. A. Lee , Y. P. Cheon , Biol. Reprod. 2022, 107, 1464. 36130223 10.1093/biolre/ioac178

[59] B. C. Paria , H. Song , S. K. Dey , Int. J. Dev. Biol. 2001, 45, 597. 11417904

[60] W. L. Cheng , P. H. Feng , K. Y. Lee , K. Y. Chen , W. L. Sun , N. Van Hiep , C. S. Luo , S. M. Wu , Int. J. Mol. Sci. 2021, 22, 12828. 34884633 10.3390/ijms222312828 PMC8657471

[61] M. Shimada , T. Umehara , Y. Hoshino , Reprod Med Biol 2016, 15, 201. 29259438 10.1007/s12522-016-0236-x PMC5715866

[62] J. M. Luo , T. T. Zhang , Y. Y. He , H. N. Luo , Y. Q. Hong , Z. M. Yang , Int. J. Mol. Sci. 2023, 24, 3163.36834576

[63] L. Zhang , W. Guo , Q. Chen , X. Fan , Y. Zhang , E. Duan , Cell Tissue Res. 2009, 338, 413. 19841944 10.1007/s00441-009-0884-9

[64] B. Li , Y. P. Yan , Y. Y. He , C. Liang , M. Y. Li , Y. Wang , Z. M. Yang , Sci. Signal. 2023, 16, eadd0645. 36853961 10.1126/scisignal.add0645

[65] Z. S. Yang , H. Y. Pan , W. W. Shi , S. T. Chen , Y. Wang , M. Y. Li , H. Y. Zhang , C. Yang , A. X. Liu , Z. M. Yang , Int. J. Mol. Sci. 2021, 23, 199.35008625

[66] T. Fu , H. T. Zheng , H. Y. Zhang , Z. C. Chen , B. Li , Z. M. Yang , FEBS Lett. 2019, 593, 2040. 31155707 10.1002/1873-3468.13468

[67] Y. J. Kang , K. Forbes , J. Carver , J. D. Aplin , Hum. Reprod. 2014, 29, 739. 24442579 10.1093/humrep/det433

[68] Z. Song , B. Li , M. Li , J. Luo , Y. Hong , Y. He , S. Chen , Z. Yang , C. Liang , Z. Yang , Int. J. Mol. Sci. 2022, 23, 3699. 35409055 10.3390/ijms23073699 PMC8998724

