# Supplementary material for: Embryo‐Derived Cathepsin B Promotes Implantation and Decidualization by Activating Pyroptosis
Source: Adv Sci (Weinh). 2024 Sep 24;11(43):2402299. doi: 10.1002/advs.202402299 (PMC11578360; doi:10.1002/advs.202402299)
Supplement: Supplementary file 1 — Supporting Information [file ADVS-11-2402299-s001.docx]

**Supplementary materials**


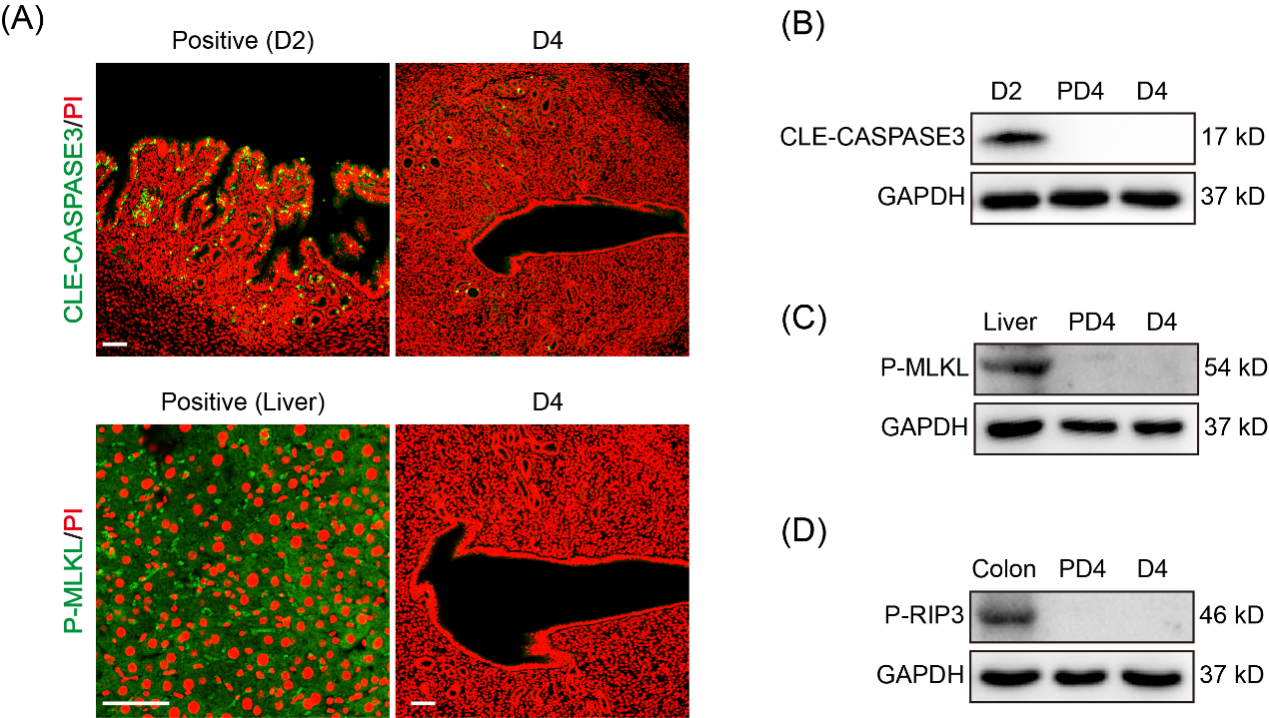


**Figure S1.** Apoptosis and Necrosis in mouse pregnant uteri. (A) Immunofluorescence of cleaved caspase 3 (green) in day 2 pregnant uterus (positive tissue) and P-MLKL (green) in mouse liver (positive tissue) and PI fluorescence (red). (B) Western blot analysis of cleaved caspase 3 in mouse uteri on days 2 and 4 of pregnancy, and day 4 of pseudopregnancy. (C) Western blot analysis of P-MLKL in mouse liver and in mouse uteri on day 4 of pregnancy and day 4 of pseudopregnancy. (D) Western blot analysis of P-RIP3 in mouse colon and in mouse uteri on day 4 of pregnancy and day 4 of pseudopregnancy. Scale bar = 250 μm.


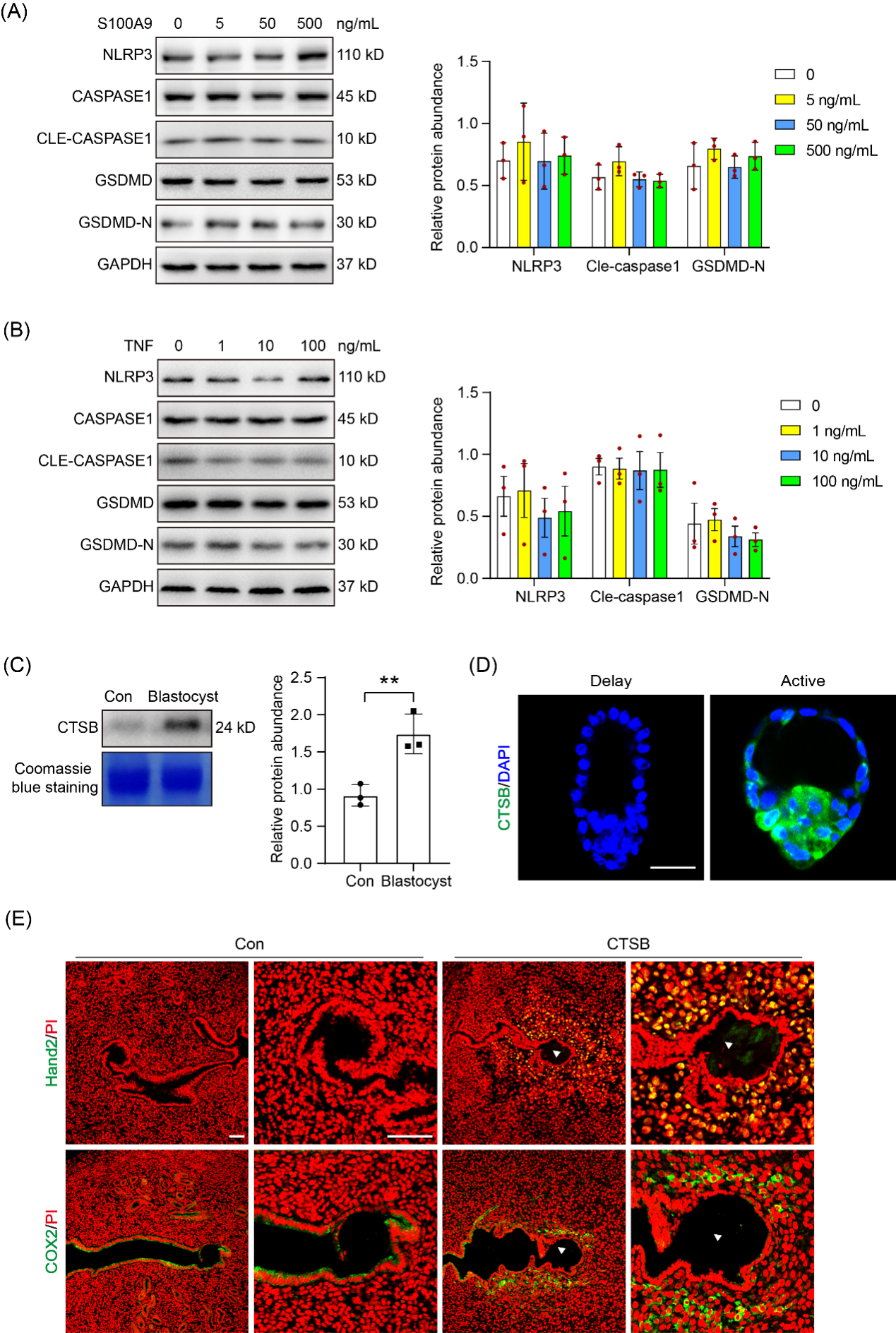


**Figure S2.** Blastocyst-derived CTSB induces pyroptosis. (A) Western blot analysis and quantification for NLRP3, CASPASE 1, cleaved CASPASE 1, GSDMD, and GSDMD-N protein levels after endometrial epithelial cells were treated with S100A9 for 3 h. (B) Western blot analysis and quantification for NLRP3, CASPASE 1, cleaved CASPASE 1, GSDMD and GSDMD-N protein levels after endometrial epithelial cells were treated with TNF for 3 h. (C) Western blot analysis and quantification of CTSB levels in cultured medium after mouse blastocysts were cultured in 20 μL KSOM for 6 h. Coomassie bright blue staining was used as a loading control (n=3 per group). (D) CTSB immunofluorescence (green) and DAPI (blue) in mouse delayed and activated blastocysts. (E) Immunofluorescence of HAND2 (green) and COX2 (green), and PI fluorescence (red) in day 5 pseudopregnant uteri after CTSB-soaked beads were transferred into uterine lumen of day 4 pseudopregnant mice. Arrowhead, CTSB-soaked beads. Scale bar = 250 μm. Data were presented as mean ± SD. **: P < 0.01, by two-tailed Student’s t-test.


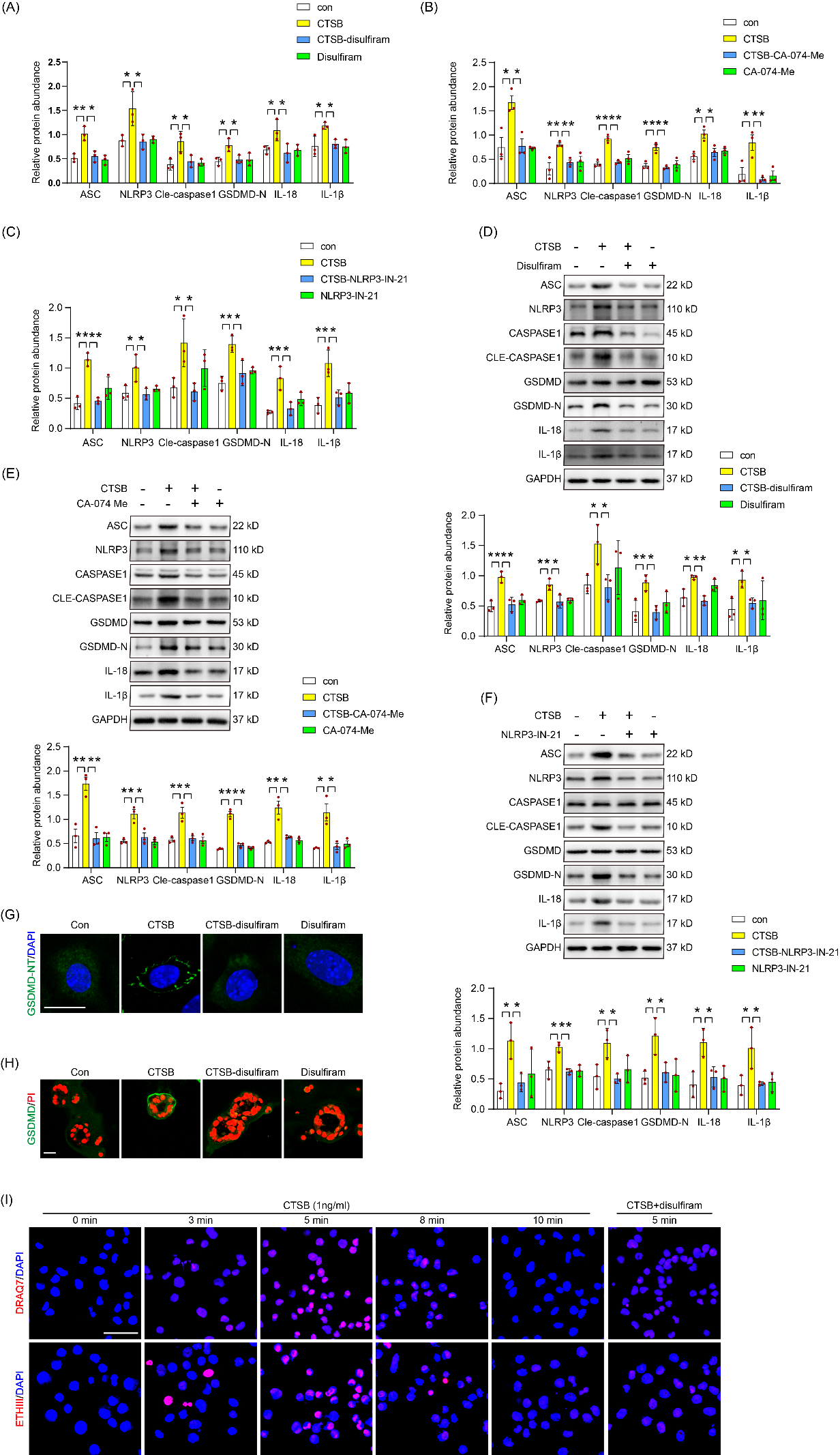


**Figure S3.** CTSB induces pyroptosis. (A) Western blot quantification of pyroptosis-associated protein levels after endometrial epithelial cells were treated with CTSB, CTSB and disulfiram, or disulfiram for 3 h. (B) Western blot quantification of pyroptosis-associated protein levels after endometrial epithelial cells were treated with CTSB, CTSB and CA-074 Me, or CA-074 Me for 3 h. (C) Western blot quantification of pyroptosis-associated protein levels after endometrial epithelial cells were treated with CTSB, CTSB and NLRP3-IN-21, or NLRP3-IN-21 for 3 h. (D) Western blot analysis and quantification of pyroptosis-associated protein levels after endometrial organoids cells were treated with CTSB, CTSB and disulfiram, or disulfiram for 3 h. (E) Western blot analysis and quantification of pyroptosis-associated protein levels after endometrial organoids were treated with CTSB, CTSB and CA-074 Me, or CA-074 Me for 3 h. (F) Western blot analysis and quantification of pyroptosis-associated protein levels after endometrial organoids cells were treated with CTSB, CTSB and NLRP3-IN-21, or NLRP3-IN-21 for 3 h. (G) Immunofluorescence of GSDMD-NT-Flag (green) and DAPI (blue) after endometrial epithelial cells transfected with pBOB-mGSDMD-NT-Flag plasmid were treated with CTSB, CTSB and disulfiram, or disulfiram for 3 h. (H) Immunofluorescence of GSDMD (green) and PI (red) after endometrial organoids were treated with CTSB, CTSB and disulfiram, or disulfiram for 3 h. (I) Fluorescence of ETHIII (red), DRAQ (red) and DAPI (blue) after endometrial epithelial were treated with CTSB for different time points. Scale bar = 125 μm. Data were presented as mean ± SD. * P < 0.05, ** P < 0.01, by two-tailed Student’s t-test.


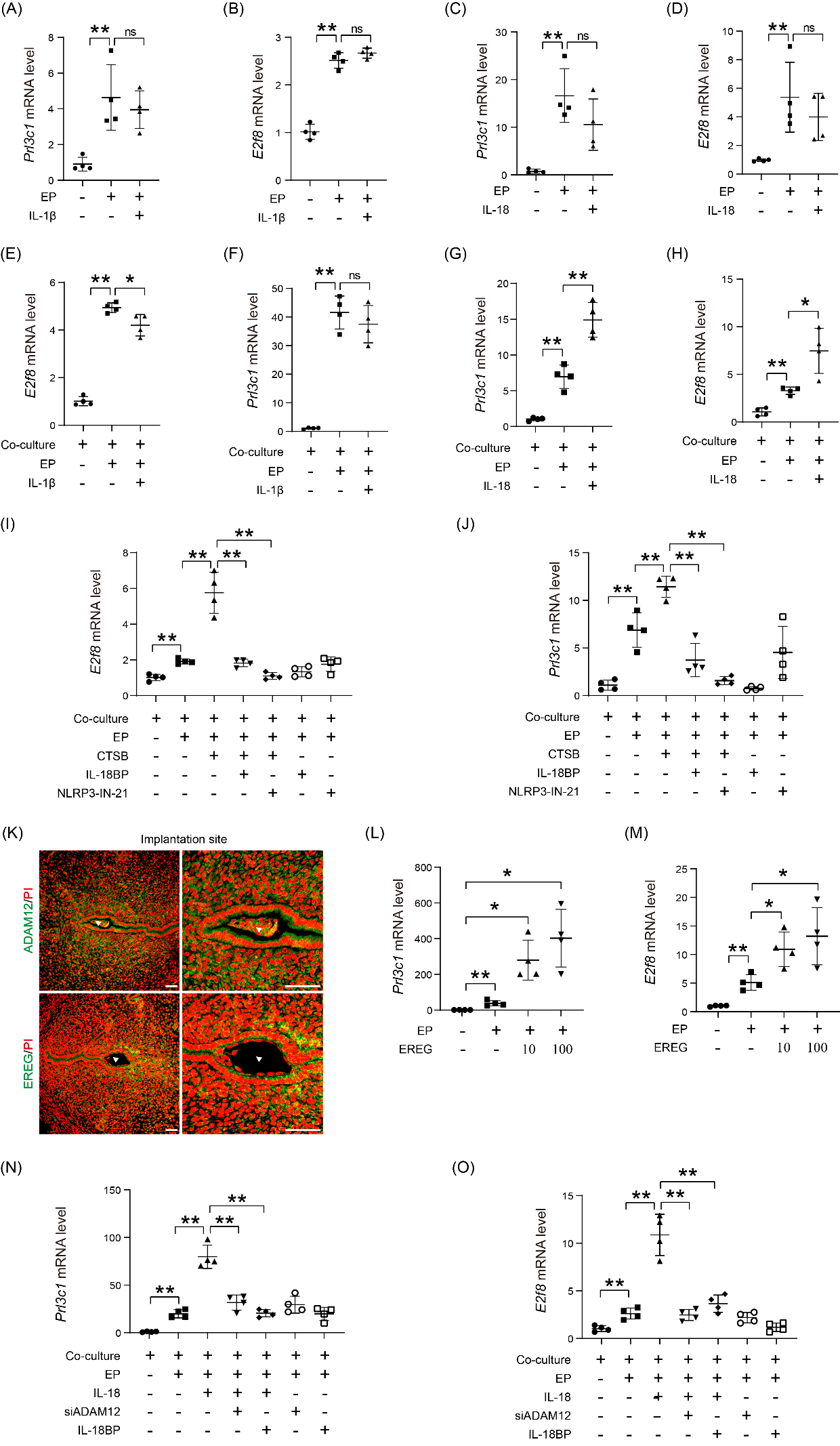


**Figure S4.** IL-18 promotes decidualization by activating ADAM12/EREG. (A) qPCR analysis on effects of IL-1β on *Prl3c1* mRNA level under in vitro decidualization for 48 h (n=4 per group). (B) qPCR analysis on effects of IL-1β on *E2f8* mRNA level under in vitro decidualization for 48 h (n=4 per group). (C) qPCR analysis on effects of IL-18 on *Prl3c1* mRNA level under in vitro decidualization for 48 h (n=4 per group). (D) qPCR analysis on effects of IL-18 on *E2f8* mRNA level under in vitro decidualization for 48 h (n=4 per group). (E) qPCR analysis on *E2f8* mRNA level in stromal cells after the co-culture of epithelial and stromal cells was treated with IL-1β for 48 h (n=4 per group). (F) qPCR analysis on *Prl3c1* mRNA level in stromal cells after the co-culture of epithelial and stromal cells were treated with IL-1β for 48 h (n=4 per group). (G) qPCR analysis on *Prl3c1* mRNA level in stromal cells after the co-culture of epithelial and stromal cells was treated with IL-18 for 48 h (n=4 per group). (H) qPCR analysis on *E2f8* mRNA level in stromal cells after the co-culture of epithelial and stromal cells were treated with IL-18 for 48 h (n=4 per group). (I) qPCR analysis on *E2f8* mRNA level after the co-culture of epithelial and stromal cells were treated with CTSB, CTSB and IL-18BP, CTSB and NLRP3-IN-21, IL-18BP, or NLRP3-IN-21 for 48 h (n=4 per group). (J) qPCR analysis on *Prl3c1* mRNA level after the co-culture of epithelial and stromal cells were treated with CTSB, CTSB and IL-18BP, CTSB and NLRP3-IN-21, IL-18BP, or NLRP3-IN-21 for 48 h (n=4 per group). (K) Immunofluorescence of ADAM12 and EREG (green) and PI (red) in mouse implantation site. Arrowhead, blastocyst. (L) qPCR analysis on *Prl3c1* mRNA level after stromal cells were treated with EREG under in vitro decidualization for 48 h (n=4 per group). (M) qPCR analysis on *E2f8* mRNA level after stromal cells were treated with EREG under in vitro decidualization for 48 h (n=4 per group). (N) qPCR analysis on *Prl3c1* mRNA level after the co-culture of epithelial and stromal cells under in vitro decidualization were treated with IL-18, IL-18 and ADAM12 siRNA, IL-18 and IL-18BP, ADAM12 siRNA, or IL-18BP for 48 h (n=4 per group). (O) qPCR analysis on *E2f8* mRNA level after the co-culture of epithelial and stromal cells under in vitro decidualization were treated with IL-18, IL-18 and ADAM12 siRNA, IL-18 and IL-18BP, ADAM12 siRNA, or IL-18BP for 48 h (n=4 per group). MEEC, mouse endometrial epithelial cell. EP, treatment with estradiol-17β and progesterone. Data were presented as mean ± SD. *: P < 0.05; **: P < 0.01; ns: not significant, by two-tailed Student’s t-test.


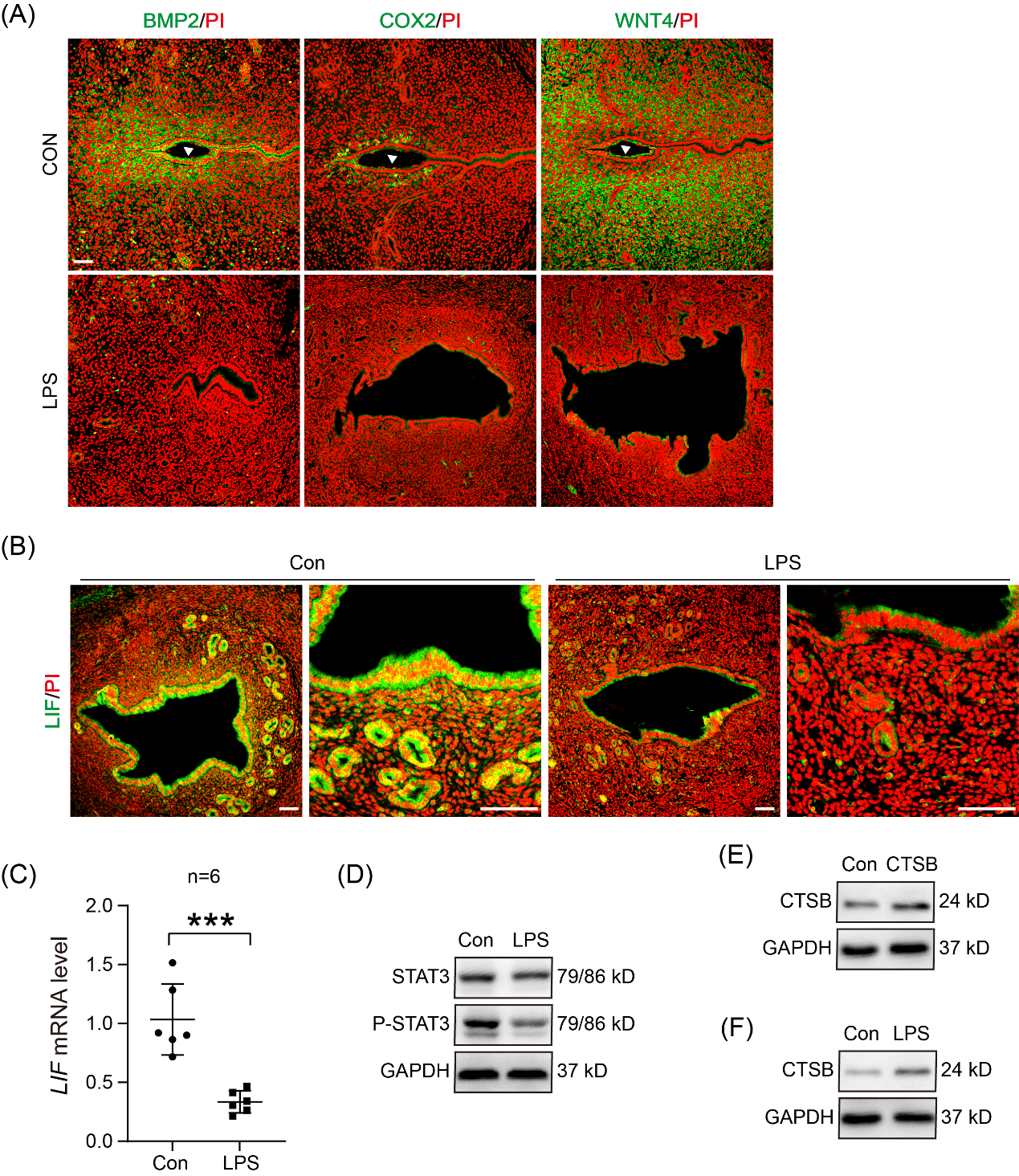


**Figure S5.** LPS inhibits implantation and decidualization. (A) Immunofluorescence of BMP2, COX2 and WNT4 (green) and PI (red) in control and LPS-treated mouse uterus. Arrowhead, blastocyst. (B) Immunofluorescence of LIF (green) and PI (red) in LPS-treated mouse uterus. (C) qPCR analysis for *Lif* mRNA level in LPS-treated mouse uterus (n=6 per group). (D) Western blot analysis for phosphorylated STAT3 in LPS-treated mouse uterus. (E) Western blot analysis of CTSB protein levels after endometrial epithelial cells were treated with CTSB. (F) Western blot analysis of CTSB protein levels after endometrial epithelial cells were treated with LPS. Scale bar = 250 μm. Data were presented as mean ± SD. ***: P < 0.001, by two-tailed Student’s t-test.


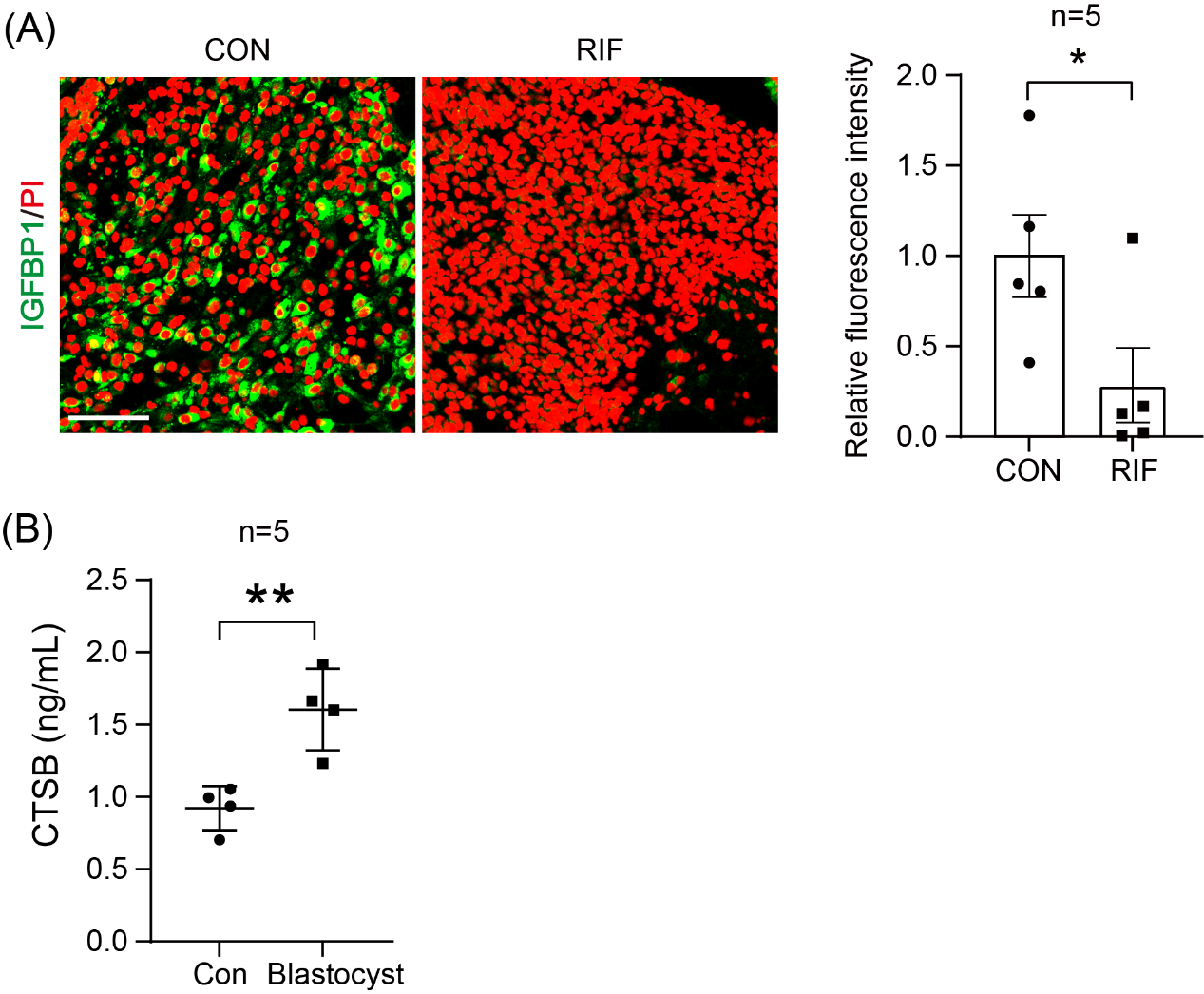


**Figure S6.** IGFBP1 immunofluorescence in mid-secretory endometrium and RIF patient's endometrium. (A) IGFBP1 immunofluorescence (green) and PI fluorescence (red) in control and RIF endometrium (n=5 per group). (B) ELISA analysis of CTSB concentrations in human blastocysts-conditioned medium (n=4 per group). RIF, recurrent implantation failure. Scale bar = 125 μm. Data were presented as mean ± SD. *: P < 0.05; **: P < 0.01, by two-tailed Student’s t-test.

**Table.1** Primer sequences used in this study were as follows:

| Primer sequences | |
| --- | --- |
| Mouse*-E2f8*-sense | GTGCTTCGTAGAACTCCCTG |
| Mouse-*E2f8*-antisense | GCAATGTCATACAGCCTCCT |
| Mouse-*Lif*-sense | AAAAGCTATGTGCGCCTAACA |
| Mouse-*Lif*-antisense | GTATGCGACCATCCGATACAG |
| Mouse*-Prl8a2*-sense | AGCCAGAAATCACTGCCACT |
| Mouse*-Prl8a2*-antisense | TGATCCATGCACCCATAAAA |
| Mouse*-Prl3c1*-sense | GCCACACGATATGACCGGAA |
| Mouse*-Prl3c1*-antisense | GGTTTGGCACATCTTGGTGTT |
| Mouse*-Rpl7*-sense | GCAGATGTACCGCACTGAGATTC |
| Mouse*-Rpl7*-antisense | ACCTTTGGGCTTACTCCATTGATA |
